# Modulating Fermentative, Varietal and Aging Aromas of Wine Using non-*Saccharomyces* Yeasts in a Sequential Inoculation Approach

**DOI:** 10.3390/microorganisms7060164

**Published:** 2019-06-06

**Authors:** Inês Oliveira, Vicente Ferreira

**Affiliations:** Laboratorio de Análisis del Aroma y Enología (LAAE), Instituto Agroalimentario de Aragón (IA2) (UNIZAR-CITA), Department of Analytical Chemistry, Faculty of Sciences, Universidad de Zaragoza, 50009 Zaragoza, Spain; inespbo@unizar.es

**Keywords:** fermentation, aroma precursors, glycosides, aroma modulation, aging, fruitiness, kerosene, floral notes, phenolic notes

## Abstract

The goal of this study is to assess to what extent non-*Saccharomyces* yeasts can introduce aromatic changes of industrial interest in fermentative, varietal and aged aromas of wine. Aroma precursors from Riesling and Garnacha grapes were extracted and used in two independent sequential experiments. Synthetic musts were inoculated, either with *Saccharomyces cerevisiae* (Sc) or with *Pichia kluyveri* (Pk), *Torulaspora delbrueckii* (Td) or *Lachancea thermotolerans* (Lt), followed by Sc. The fermented samples were subjected to anoxic aging at 50 °C for 0, 1, 2 or 5 weeks before an aroma analysis. The fermentative aroma profiles were consistently changed by non-*Saccharomyces*: all strains induced smaller levels of isoamyl alcohol; Pk produced huge levels of aromatic acetates and can induce high levels of fatty acids (FA) and their ethyl esters (EE); Td produced large levels of branched acids and of their EE after aging, and induced smaller levels of FA and their EE; Lt produced reduced levels of FA and their EE. The varietal aroma was also deeply affected: TDN (1,1,6-trimethyl-1,2- dihydronaphthalene) levels in aged wines were reduced by Pk and enhanced by Lt in Garnacha; the levels of vinylphenols can be much reduced, particularly by Lt and Pk. TD and Lt can increase linalool and geraniol in young, but not in aged wines.

## 1. Introduction

The controlled use of non-*Saccharomyces* yeasts as a promising way to improve the sensory diversity of wines was first proposed in the nineties [1,2,3]. In early research, *Candida stellata* was suggested to increase the glycerol content of the wines, with the understanding that this compound was an active contributor to the in-mouth sensory properties of wine. Soon, other researchers demonstrated that *Pichia anomala* was able to produce high levels of acetate esters [4,5], particularly of isoamyl and phenylethyl acetates, compounds which in combination are able to induce pear-like, sweet-banana and floral notes in wine aroma. This ability to produce acetates was later advantageously used to increase the levels of the powerful 3-mercaptohexyl acetate (odor reminding of passion fruit), at the expense of the less powerful 3-mercaptohexanol (green, grapefruit, and rocket salad) by the co-fermentation of *Pichia kluyveri* (Pk) with *Saccharomyces cerevisiae* [6] (Sc). However, other authors have reported far more modest increases of acetates when using a sequential fermentation approach with a Sc strain with a high tendency to form acetates [7].

The effects of *Torulaspora delbrueckii* (Td) are far more subtle. Various authors have reported a positive effect on taste and aroma, but the chemicals responsible for such positive effects, other than a reported ability to reduce the wine acetic acid, acetaldehyde and ethyl acetate contents, are not clear [8,9]. This strain was used in semi-industrial experiments [10,11] demonstrating that it is able to produce sensorially different wines. However, the analytical reasons for those sensory changes were again not clear, since the contents of most of the measured odor-active compounds were just marginally affected by the presence of non-*Saccharomyces* [10,11]. Similar conclusions were derived from data reported by other authors [12]. Moreover, some studies even claiming a positive effect on the sensory profile, report strong significant decreases in the levels of isoamyl acetate and medium chain fatty acid ethyl esters [13]. In another report, the highest aroma quality obtained by a Td strain (NSA-1) in sequential fermentation can only be related to its smallest levels of isoamyl alcohol [14], which is a known fruity suppressor [15], but not to any higher content of fruity odorants. These last reports also confirm that some of the effects are highly strain-dependent. In contrast, in the case of cherry wines, the sequential fermentation of Td (Zymaflore, Alpha) showed increased levels of relevant ethyl esters (ethyl butyrate, ethyl isovalerate, ethyl hexanoate, octanoate and decanoate) and also slightly higher levels of linalool [16].

The effects of Td on the production of fruity esters in wine were specifically addressed in a more recent work [17]. The authors conclude that ethyl propanoate, ethyl isobutyrate and ethyl dihydrocinnamate can be considered specific markers of the fermentation activity of Td, while isobutyl and isoamyl acetates increased in mixed fermentations as the likely result of positive interactions between Td and Sc. Interactions are obviously favored when both yeasts are simultaneously inoculated, while sequential inoculation favors the development of the specific markers [17]. These authors specifically demonstrated that the addition of ethyl propanoate, ethyl isobutyrate and ethyl dihydrocinnamate at the levels found in excess in the wine made by the sequential fermentation with Td, to the wine made with Sc, significantly increased fruitiness and complexity. Out of the three odorants, in relation to odor thresholds, ethyl isobutyrate is the most powerful. This ethyl ester is usually produced below the stoichiometric proportion corresponding to the esterification equilibrium (isobutyric acid + ethanol ←→ ethyl isobutyrate). As the isobutyric acid levels were not measured, it is not possible to foresee how the differences will evolve with time. Other authors, using Td with a killer character, did confirm higher levels of ethyl propanoate, but in their case Td wines were less fresh and intense, developing dry/cooked fruit and pastry/candy notes, which could not be attributed to any analyzed aroma compound [18]. It can be hypothesized that such a character may be related to an increased methional content, since Td has been reported to consistently produce higher levels of methionine derivatives [19].

Regarding *Lachancea spp*, the most studied species is *L. thermotolerans* (Lt), which can be naturally found in wine fermentations [20] and which has been proposed by numerous authors because of its ability to produce lactic acid and hence increase the wine total acidity and decrease the wine pH [21,22,23,24,25,26,27]. The reported effects on the aroma composition are the decrease of isobutanol and isoamyl alcohol [7,24,26], or of isoamyl alcohol alone [27], which is a positive factor, and a strong decrease of ethyl esters and acetates, which is less favorable for making fruity wines [24,26]. This last decrease is not observed at all in some other reports [7] or it is observed only in isoamyl acetate, but not for phenylethyl acetate [27,28]. Some of these discrepancies could be attributed to the strong modulating role played by oxygen in the dynamics of the fermentation and in the profile of the produced aroma volatiles [29], while some others could be attributed to differences between strains. For instance, a recent report reveals that the Y940 strain produced smaller levels of isoamyl alcohols than the Sc control, while the Concerto strain produced higher levels [28].

In spite of the fact that it has been known that some non-*Saccharomyces* yeasts have potentially higher glycosidic activities [30], not many papers deal with the ability of these yeasts to produce aroma compounds from glycosidic precursors. As many aroma glycosides are located in the grape skin, other enzymatic activities, such as pectinase, cellulose, xylanase and glucanase, may contribute to enrich aroma compounds derived from grape glycosidic precursors. It has been described that Pk displays strong exocellular glucanase and pectinase activities [31], but it does not seem to have glucosidase activity [32]. As for Lt, it has all these activities, although it displays glucosidase at a low relative intensity (6%). On the other hand, Td had high levels of pectinase and low levels of cellulase, but not glucosidase [32]. A recent report about different *Lachancea* strains, including Lt, confirms the existence of β-glucosidase activity, specifically in the cell wall, reaching a maximum activity on the second day in monocultures, or on the sixth day in mixed cultures [28]. The report also reveals relatively large differences between different strains.

Similarly, information about the potential effect of non-*Saccharomyces* yeasts on these varietal aroma compounds in real vinifications is limited and somewhat contradictory. In the case of Shiraz, the different non-*Saccharomyces* yeast used in sequential fermentations produced higher levels of linalool, geraniol and other less relevant terpenes than the Sc that was used to complete the fermentation [33]. In Riesling, the levels of linalool and β-damascenone were not changed by any of the non-*Saccharomyces* that were studied (Lt, Pk and *Metschnikowia pulcherrima*) [7]. In Muscat, Lt in fact produced significantly smaller levels of linalool and similar or just slightly higher levels of geraniol than the Sc control [28].

One usual limitation of most of the previous studies is that the analysis of the aroma compounds is carried out at a single time point, while many aroma compounds can evolve with aging time in different ways. As previously mentioned, ethyl esters of branched acids tend to increase continuously with aging time, while acetate esters tend contrarily to hydrolyze quickly. This implies that conclusions regarding the ability of yeasts to produce aromas may depend on the time of analysis. In this regard, some of the most important varietal aroma compounds, such as linalool and geraniol, are quite unstable in terms of the wine pH so that their levels quickly decay. However, the decay functions will largely depend on the remaining pool of un-hydrolyzed precursors, so that a too large β-glucosidase activity during fermentation may in fact produce a very aromatic young wine with an extremely short shelf-life. However, another grape-derived important aroma compound, trimethyl-dihydronaftalene (TDN), responsible for the kerosene off-odor identified in Riesling wines, requires long aging times to accumulate, and hence assessing the specific action of yeast requires analyzing old wines.

Taking all this into account, the main goal of the present paper is to assess the ability of three commercial non-*Saccharomyces* strains (Pk, Lt and Td) in a sequential inoculation approach, to introduce aroma changes of industrial interest in fermentative, varietal and aged aromas of wines.

## 2. Material and Methods

### 2.1. Reagents and Standards

Dichloromethane (DCM), ethanol and methanol (≥99%) Disto-Pesticide residue grade were supplied by Merck (Darmstadt, Germany). Milli-Q^®^ system was supplied from Millipore (Merck, Darmstadt, Germany). 2-butanol (≥99%), 4-methyl-2-pentanol (99%), 4-hydroxi-4-methyl-2-pentanone (99%), ethyl heptanoate (99%) and heptanoic acid (99%) were used as internal standards for the major compounds analysis, and 2-octanol (99.5%), 3-octanone (99%) and 3,4-dimethylphenol (99%) were used as internal standards for the minor and trace compounds analysis and were purchased from Merck. The chemical standards used in this study were supplied by Merck with a purity of >98%. TDN was synthesized by Synchem UG &Co (Felsberg, Germany) with a purity of 80%.

### 2.2. Glycosidic Precursors Extraction

The glycosidic precursor fractions were from a 2016 harvest of approximately 23 kg of grapes of each variety. Riesling grapes were obtained in Neustadt an der Weinstrasse, Germany and Garnacha grapes were given by Bodegas Román from D.O. Campo de Borja, Spain. The grapes were crushed by feet, added with 60 mg/L of SO_2_ and cold macerated for 24 h in the case of Riesling and 48 h in the case of Garnacha. The maceration took place in the presence of pectolytic enzymes -Lafazym CL (Laffort, Bordeaux, France) to facilitate racking. The Riesling grapes were pressed using an Europress (Scharfenberger, Bad Dürkheim, Germany) and then cleared by flotation with N_2_ and divided into three 5 L batches with an addition of 80 mg/L of SO_2_ in each. The Garnacha grapes were pressed using a manual hydraulic press and then cleared by sedimentation. The cleared must was divided into two 5L batches with the addition of 80 mg/L of SO_2_ in each.

The precursors were extracted with 5 g of Lichrolut-EN resins, previously washed and preconditioned (packing them in 60 mL cartridges) with 45 mL of dichloromethane, 45 mL of methanol and 54 mL of Milli-Q^®^ water. The resins were then let freely in the must with constant agitation during 48 hours, in a cool room. After the extraction, the resins were recovered using a paper filter with 150 mm pores (Macherey-Nagel, Düren, Germany), rinsed with Milli-Q^®^ water, repacked in the cartridges and dried in a VAC ELUT station (Varian, Palo Alto CA, USA). The resin was washed with 45 mL of dichloromethane to remove grape aroma compounds and was further eluted with 90 mL of ethyl acetate with 10% methanol (*v*/*v*). Afterwards, the resins were re-activated, and the extraction process was repeated adding 50 mg/L of SO_2_ to each must batch. The eluted fractions were mixed and dried from their solvent using a rotavapor R-215 coupled with a heating bath B-491, from Buchi (Flawil, Switzerland) and N_2_ flow. Prior to usage, the glycosidic fractions were re-dissolved with 70 mL of ethanol.

### 2.3. Synthetic Must

A complex synthetic must was prepared and adjusted to 3.5 pH and then filtered by means of sterile cellulose nitrate membrane filters with 0.45 µm pores (Albet, A&S Filter Co., Ltd., Dassel, Germany) inside a vertical laminar flow chamber PV-100 (Telstar S.A, Barcelona, Spain) to ensure that all manipulations were done under aseptic conditions. All of the glassware was sterilized using an autoclave AES-28 from Raypa (Barcelona, Spain). The must composition was adapted from Bely et al., [34] and had the following composition: oligoelements: MnCl_2_•4H_2_O 4.7 mg/L, Co(NO_3_)_2_•6H_2_O 0.49 mg/L, NaMoO_4_•2H_2_O 0.19 mg/L, CuCl_2_ 0.54 mg/L, KIO_3_ 1.29 mg/L, H_3_BO_3_ 1 mg/L; malic acid 0.3 g/l; tartaric acid 3 g/L; SO_4_Mg•7H_2_O 0.2 g/L; KH_2_PO_4_ 2 g/L; CaCl_2_•2H_2_O 0.155 g/L; citric acid 0.3 g/L; vitamins from Merck (≥98%): pyridoxine hydrochloride 1 mg/L, nicotinic acid 1 mg/L, calcium panthothenate 1 mg/L, thiamine hydrochloride 1 mg/L, ρ-aminobenzoic acid 1 mg/L, riboflavin 0.2 mg/L, folic acid 0.2 mg/L, biotin 0.04 mg/L. Myo-inositol 0.3 g/L; and ergosterol 15 mg/L. Sugars were obtained from Panreac Applichem (Barcelona, Spain): glucose 100 g/L and fructose 100 g/L. Tween80^®^ 0.05% (*v*/*v*) (Merck). Nitrogen source: (NH_4_)2HPO_4_ 219.9 mg/L; amino acids (Merck) (mg/L): GABA 44.37, alanine 58.51, tyrosine 14.34, valine 17.73, isoleucine 14.43, leucine 13.42, aspartate 34.82, glutamic acid 61.83, glutamine 104.83, serine 21.21, glycine 1.11, histidine 109.2, threonine 18.8, arginine 199.5, proline 241.46, methionine 29.85, phenylalanine 11.15, and lysine 3.33. The synthetic must was freshly prepared after each experiment.

### 2.4. Fermentation

Four commercial yeasts were selected from the Chr. Hansen (Hørsolm, Denmark) Viniflora^®^ product line to ferment the synthetic must: *Saccharomyces cerevisiae* (Merit.ferm), *Pichia kluyveri* (FrootZen^TM^), *Torulaspora delbrueckii* (Prelude^TM^) and *Lachancea thermotolerans* (Concerto^TM^). With the exception of Pichia kluyveri (FrootZen^TM^), which is commercially available for direct inoculation, the remaining strains are dried products. As the fermentations with the different varieties were not carried out at the same time, after the product opening and usage for the first experimental setup, the yeast cells were preserved at −80 °C. For that, all of the dried yeasts were cultured, and pure colonies were grown in a yeast extract peptone dextrose medium (YEPD). The yeast cells were aliquoted (10^7^ CFU/mL) and preserved with glycerol at −80 °C. The inoculations for the Garnacha fermentations were made from the dried products following the product instructions: 1 g of dried yeast from a recently opened package was hydrated in 20 mL of water for 1 hour using a water bath at 35 °C to maintain the temperature. Afterwards, 20 mL of synthetic must was added to pre-activate the cells at the same temperature for 30 min. The fermenters were inoculated with 10^6^ cells/mL. For the Riesling experimental fermentations, the inoculations were made from the frozen preserved cells: each aliquot was centrifugated, and the pellet was washed with a phosphate buffer solution. A clean pellet was used to inoculate each fermenter with 10^6^ CFU/mL.

Two series of 8 fermenters containing 350 mL of synthetic must were prepared using 500 mL blue cap glass flasks (Ilmabor TGI, Ilmenau, Germany). One series was spiked with the glycosidic precursor fraction, and the second one was used as the must control. The four yeast strains that were selected were used to ferment both series, with glycosidic precursors and controls, following the same inoculation protocol. Two biological replicates of each experiment were prepared. Fermentations with only *S. cerevisiae* were used as the microbiologic controls while all non-*Saccharomyces* were sequentially inoculated with *S. cerevisiae* after 4 days of individual fermentation. The fermenters were closed with airlock valves, and the fermentations were carried out at 20 °C. The fermentation progress was followed by weighing the fermenters on a daily basis until the weight loss between two consecutive measurements was smaller than 0.1 g.

Once fermentation was finished, the fermenters were centrifuged and aliquoted for the different analysis inside an anoxic chamber Jacomex (Dagneux, France). Additionally, the wines were aliquoted into air tight Wine In Tubes (WIT, Bordeaux, France), bagged in high density plastic bags containing oxygen scavengers AnaeroGenTM (Thermo Scientific, Waltham, MA, USA) and aged at 50 °C in a laboratory heater (J.P. Selecta, Barcelona, Spain). The samples were collected and analyzed at the end of the fermentation and after 1, 2 and 5 weeks of accelerated aging.

Unfermented controls were included to assess the effect of acid hydrolysis on the release and formation of volatile compounds. Synthetic wine with 12% ethanol, 3.5 g/L of tartaric acid and Milli-Q^®^ water was adjusted to pH 3.5. Eight WIT tubes (WIT France, Blanquefort, France) were prepared inside the anoxic chamber: four WIT were capped with only synthetic wine, and in the remaining four WIT the synthetic wine was spiked with the precursor fraction. The WIT were further bagged as described for the accelerated aging experiments. All eight WIT were kept in the same incubators as the fermented incubators (21 °C) and then the accelerated aging samples (50 °C). Two tubes, one with only synthetic wine and one spiked with precursors, were taken after fermentation and after 1, 2 and 5 weeks at 5 °C.

### 2.5. Analytical Methods

The wines were characterized according to their general enological parameters using the recommended methodologies by OIV (International Organization of Vine and Wine, 2011 edition) for reducing the sugars, pH, total acidity and volatile acidity, and free and total SO_2_.

#### 2.5.1. Analysis of Major Volatile Compounds

Analyses of higher alcohols, volatile fatty acids and major esters were carried out by liquid-liquid microextraction with DCM, followed by a GC analysis with Flame Ionization Detection (FID), following the methodology proposed by Ortega et al. [35]. Briefly, 3 mL of wine previously spiked with the internal standard solution are diluted with 7 mL of Milli-Q water, added with 4.1 g of (NH_4_)_2_SO_4_, and then extracted with 0.25 mL of DCM using 90 min of horizontal agitation. The tubes are further centrifuged (2500 rpm for 10 min) to recover the organic phase with a syringe. This phase is further analyzed by GC-FID.

#### 2.5.2. Analysis of Minor and Trace Volatile Compounds

Aroma compounds were extracted by a solid phase extraction (SPE) using the methodology described by Lopez et al., [36] with some modifications. 15 mL of wine was spiked with three internal standards (2-octanol, 3-octanone and 3,4-dimethylphenol) and percolated through an SPE cartridge packed with 65 mg of LiChrolut-EN resins previously activated with 2 mL of DCM, methanol and hydro-alcoholic solution with 12% ethanol. The resin was washed with a solution of water with 30% methanol (*v*/*v*) and 1% of NaHCO_3_ (*m*/*v*) and dried, and the aroma compounds were further eluted with 0.6 mL of DCM containing 5% methanol (*v*/*v*).

The GC-MS system was a QP2010 from Shimadzu (Quioto, Japan). The column was a DB-WAXetr from Agilent (Santa Clara CA, USA), 30 m × 0.25 mm with a 0.5 µm film thickness, preceded by a 2 m × 0.25 mm uncoated precolumn. The carrier gas was He at 1.26 mL/min. The chromatographic oven was initially at 40 °C for 5 min, the temperature was increased at 1 °C/min until 65 °C and then at 2 °C/min until 220 °C, and was kept for 50 min. An SPL injector (split/splitless) was used at a temperature of 250 °C. The injection was carried out in split-less mode; 2 µL of the extract were injected using a pressure pulse to ensure a column flow of 4.50 mL/min during 1.5 min, the time at which the split valve was opened. The temperature of the ion source was kept at 220 °C and the interface at 230 °C. The mass analyzer was set in single ion monitoring mode (SIM), and the complete list of *m*/*z* ratios selected for each compound as well as their retention times are shown in the supplementary material Table S1. Quantification was done by interpolating the SI-normalized peak area in the straight lines built by the repeated analysis of calibrated solutions containing at least three different concentration levels of each compound covering the range of occurrence. For vitispirane and Riesling acetal, for which no commercial standard was available, the identification was based on their reported mass spectra and retention indexes.

The data processing was made using Microsoft Excel Visual Basic for application (VBA) simple coding. The analysis of variance (ANOVA) was made on the compounds with the area above the limit of quantification, assessing the factors of the presence of precursor fraction, yeast strain and accelerated aging time, as well as the binary interactions (presence of precursors × yeast strain and yeast strain × aging time). The Principal Component Analysis and Scatter plots were used to analyze the data to assess the hierarchy of factors affecting the aroma formation. These analyses were performed using XLSTAT software (2018 version, Addinsoft, Long Island City, NY, USA).

## 3. Results

### 3.1. General Overview

**Figure 1:**
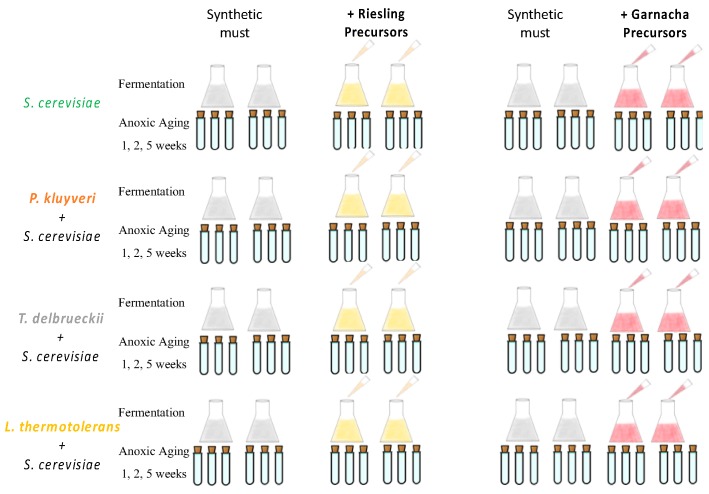
Experimental set-up: two independent sequential fermentation experiments with synthetic must were prepared. In the first experiment, one of the series was spiked with glycosidic precursors extracted from Riesling, and in the second experiment with precursors extracted from Garnacha grapes. Each experiment included a control with synthetic must, the series containing precursors, and a third series of synthetic wine spiked with precursors (not represented in the figure). The two first series were divided into four lots; one was inoculated with *S. cerevisiae*, and the others were firstly inoculated with *P. kluyveri*, *T. delbrueckii or L. thermotolerans* and, after 4 days, with the same S. cerevisiae strain. The fermented samples and unfermented controls were further aged 1, 2 and 5 weeks at 50 °C in strict anoxic conditions.

In order to assess the role of non-*Saccharomyces* yeasts on the aroma composition of young and aged wine, two parallel experiments were carried out, as schematized in Figure 1. In each experiment, synthetic must containing or not containing glycosidic aroma precursors extracted from Riesling (first experiment) or from Garnacha (second experiment) grapes was fermented either with one Sc yeast strain or with 3 different non-*Saccharomyces* following a sequential inoculation approach. The wines were further subjected to an accelerated anoxic aging (AA) in order to let the varietal and fermentative aroma develop and evolve. Three aging points (after 1, 2 and 5 weeks), in addition to the recently fermented sample, were prepared. In total, 56 different samples were produced in each experiment: 2 (with/without precursors) × 2 (replicates) × 4 (yeasts) × 4 (aging times) + 2 (1 unfermented control with precursors + 1 blank) × 4 (aging times). In total, 42 relevant aroma components were determined by GC and GC-MS in all of the samples. The complete data set is given in the supplementary material, together with the results of the statistical analysis (Tables S2–S5).

Some basic parameters of the fermented samples are shown in Table 1. The fermentation curves are given in the supplementary material (Figure S1). The fermentation took place in shorter times in the first experiment, whose samples ended with smaller levels of residual sugars, volatile acidity, and of total acidities, as seen in Table 1. In general, the samples from the first batch contained smaller levels of fermentative aroma compounds. This batch effect has some influence on the relative effects played by the different strains. For instance, the samples fermented initially by Lt and Td in the first experiment have decreased levels of volatile acidity in comparison to the samples fermented only by Sc, but the opposite is observed in the second experiment (Table 1). These effects will be taken into account in the discussion of the results.

The comparison between the samples containing precursors and those which do not, makes it possible to classify the aroma compounds into fermentative and varietal categories. Regarding the fermentative aroma compounds, 26 different aroma compounds were quantified: 6 alcohols, 8 ethyl esters, 3 acetates, 6 acids and 3 γ-lactones. Moreover, the sixteen varietal aroma compounds, exclusively found in the samples containing precursors, belonged to four different chemical classes: norisoprenoids (1,1,6-trimethyl-1,2-dihydronaphthalene –TDN-, β-damascenone, vitispirane and Riesling acetal), terpenols (linalool, geraniol and α-terpineol), volatile phenols (4-vinylguaiacol, 4-vinylphenol, guaiacol, 2,6-dimethoxyphenol and E-isoeugenol) and vanillin-derivatives (vanillin and acetovanillone). As expected, the differences between the grape varieties were only of a quantitative nature, with no existing aroma component being present in a single variety. Terpenols were more concentrated in Riesling, while the samples containing Garnacha precursors had higher levels of volatile phenols. The effects of the yeast strain on the contents and evolution of the fermentative and varietal aroma compounds will be discussed separately. The discussion will be mainly centered on those aroma compounds with a higher sensory relevance.

### 3.2. Fermentative Aroma Compounds

**Figure 2:**
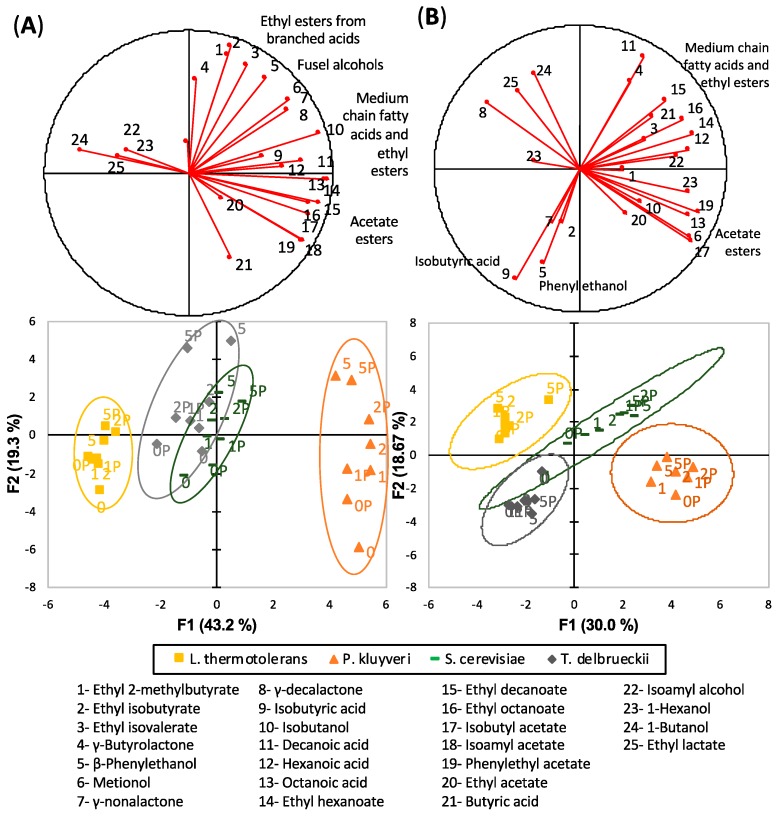
Principal component analysis carried out with fermentative aroma compounds quantified in (**A**) Riesling and (**B**) Garnacha from all of the fermented samples. The plot shows the projection of variables (top plot) or samples (bottom)—the average of two biological replicates—in the plane formed by the first two components, which retained 62.5% and 48.7% of the original variance, respectively. The numbers in the samples refer to the weeks of anoxic storage at 50 °C, and ‘P’ refers to the presence of precursors.

Figure 2 shows the distribution of the samples in the PCA plots obtained by exclusively processing data from fermentative compounds in both varieties. As can be seen, the main factor determining the position of the samples in the PCA plots in both cases is yeast, and the samples are distributed in relation to this criterion, following a common pattern in both experiments. The samples fermented exclusively with Sc are in the center, while the samples in which the first part of the fermentation was carried out by Pk are in both experiments in the right part of the plot. On the other hand, the samples inoculated first with Lt are in the left, while those made with Td were the least dissimilar to the Sc controls. The volatiles determining the differences between yeasts are, on the one hand, ethyl lactate and butanol, which in both cases are found at the highest levels in the samples made with Lt, and, on the other hand, acetate esters and ethyl esters of fatty acids; which are the maxima in the samples made with Pk.

As seen in the variable loading plots, the batch effect affected the relative positions in the plots of the ethyl esters of the branched acids (codes 1–3), β-phenylethanol (code 5), isobutyric acid (code 9) and γ-decalactone (code 8). These differences are responsible for the apparently different effect of the aging time in both plots. In the PCA from the first experiment, the aged samples had, in all cases, higher scores on the second component, regardless of the yeasts carrying out the fermentation, because, as seen in the variable loadings plot, aging leads to an increase in the ethyl esters of the branched acids levels—correlated to the 2nd component and a decrease in acetate esters –negatively correlated to this component. In the second experiment, however, aging is not so well represented because of the differential position of the ethyl esters of the branched acids and of isobutyric acid. The presence of precursors does not result in evident effects on the positioning of the samples in the plots.

### 3.3. Acetates of Higher Alcohols

**Figure 3:**
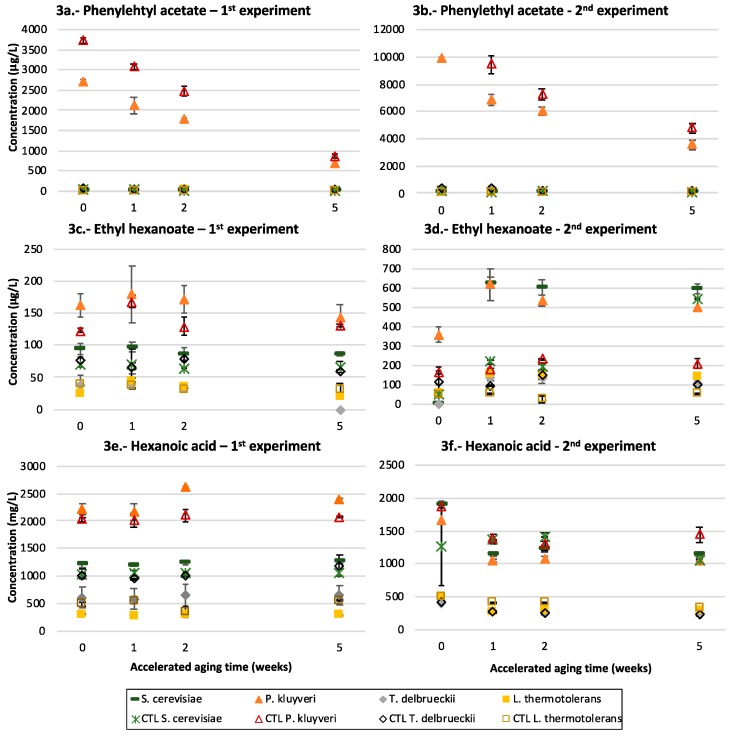
Acetate esters, fatty acids and their ethyl esters: evolution of (**a**,**b**) phenyl acetate, (**c**,**d**) ethyl hexanoate and (**e**,**f**) hexanoic acid in the Riesling and Garnacha experiments after 1, 2 and 5 weeks of accelerated aging and according to the yeast strain that carried out the fermentation: *S. cerevisiae* or *P. kluyveri, T. delbrueckii* and *L. thermotolerans* sequentially inoculated with *S. cerevisiae*. The samples without precursors are indicated as CTL.

The samples fermented first with Pk have much higher contents of the acetates of the higher alcohols (isobutyl acetate, isoamyl acetate and phenylethyl acetate), as can be clearly seen for the case of phenylethyl acetate in the plots shown in Figure 3a,b. The plots also show that the contents of this acetate decrease with time.

### 3.4. Medium Chain Fatty Acids and Their Ethyl Esters

The samples first inoculated with Pk also contained increased levels of medium chain fatty acids and of their ethyl esters, although in this case, the differences were not so outstanding as those observed for the acetates, as seen in the plots given in Figure 3c–f, for the particular case of ethyl hexanoate and hexanoic acid. It can also be observed that, in this case, the batch effect affects the relative position of the samples only fermented with Sc. In the first experiment, Sc produced levels of both the acid and the ester intermediate between the maxima observed in the samples fermented by Pk, and the minima observed for the samples fermented by Td and Lt. In the second experiment, however, the levels produced by Sc were similar to those produced by Pk. It can also be appreciated that in the second experiment there is a clear effect resulting from the presence of the precursor fraction, which in all cases except for Td contained higher levels of the ester, but not of the acid. In other words, in this batch the esterification ratio was significantly higher in the presence of the precursor fraction, except in the case of Td. As seen in the plots, these compounds remain relatively stable with time.

### 3.5. Ethyl Esters of Branched Acids

**Figure 4:**
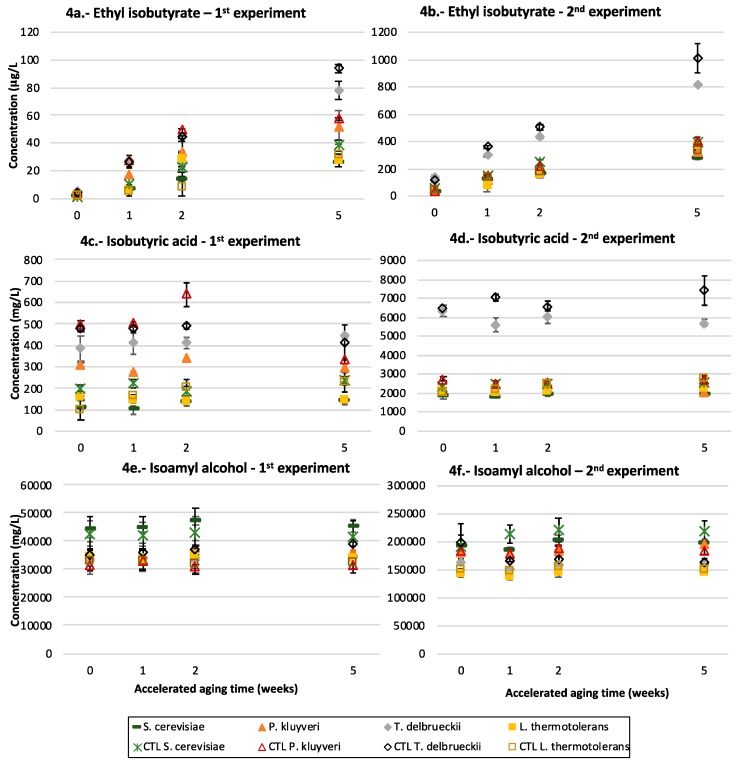
The branched acids and their ethyl esters and isoamyl alcohol: the evolution of (**a**,**b**) ethyl isobutyrate, (**c**,**d**) isobutyric acid and (**e**,**f**) isoamyl alcohol in the Riesling (1st experiment) and Garnacha (2nd experiment) experiments after 1, 2 and 5 weeks of accelerated aging and according to the yeast strain that carried out the fermentation: *S. cerevisiae* or *P. kluyveri*, *T. delbrueckii* and *L. thermotolerans* sequentially inoculated with *S. cerevisiae*. The samples without precursors are indicated as CTL.

The third family of fruity esters, the ethyl esters of the branched acids, follows a completely different aging pattern, as can be seen in Figure 4a–d for the particular cases of ethyl isobutyrate and isobutyric acid. The levels of these esters progressively increase during aging. As seen in the figures, there is a strong influence of the yeast strain on the levels of both the acid and the ester, but there is also an evident batch effect in the specific case of Pk. In both experiments, the samples initially inoculated with Td contained significantly higher levels of both compounds, but in the first batch, the levels in the samples fermented by Pk were close to those measured in the samples fermented by Td. There is also a notable and negative effect of the presence of the precursors in both batches for Td and Pk. The same results are observed for the other important esters in this group: ethyl 2-methylbutyrate and ethyl isovalerate.

### 3.6. Isoamyl Alcohol

The results for this compound can be seen in Figure 4e,f. As can be seen, in both experiments all the fermentations in which non-Saccharomyces yeast participated produced smaller levels of the alcohol.

### 3.7. Varietal Aroma Compounds

**Figure 5:**
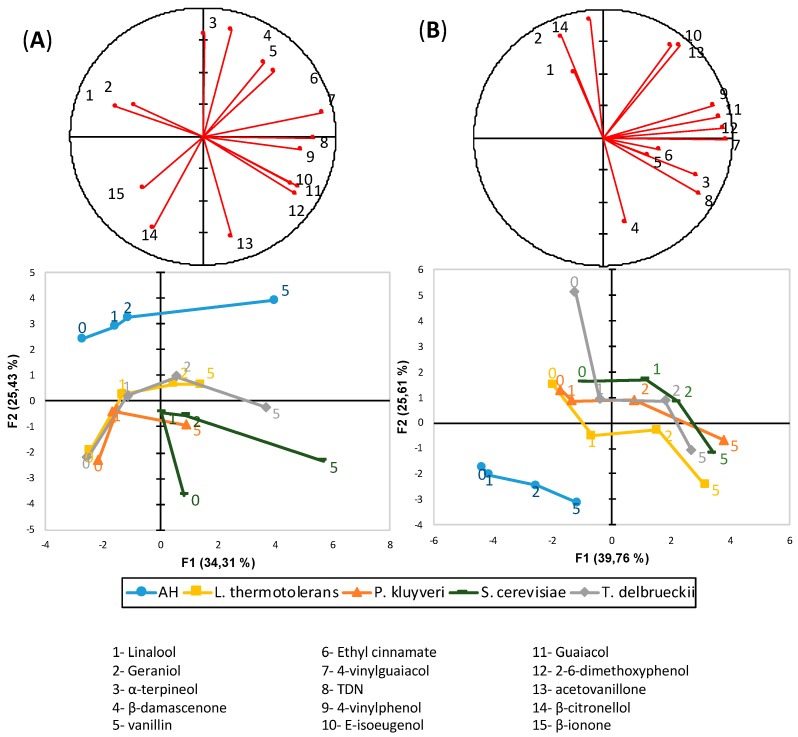
The principal component analysis carried out with the varietal aroma compounds quantified in (**A**) Riesling and (**B**) Garnacha from all of the fermented samples. The plot shows the projection of the variables (top plot) or samples (bottom) average of two biological replicates—in the plane formed by the first two components, which retained 59.74% and 65.37% of the original variance, respectively. The numbers in the samples refer to the weeks of anoxic storage at 50 °C, and each strain pattern is highlighted with a line.

Regarding the varietal aroma, Figure 5 shows the two principal component plots corresponding to the PCA analysis carried out with the data from the varietal aroma compounds in the two experiments. As can be seen, the most obvious factor dominating the sample distribution in the plots is the existence of fermentation, since in both cases the unfermented controls containing just the precursors in the synthetic wine are clustered apart. This confirms that the fermentation exerts a powerful effect on the varietal aroma composition. As can be read from the variable loading plots, the differences are due to a generally higher level of aroma compounds in the fermented samples, particularly of volatile phenols and vanillin-derivatives. The factor explaining the sample distribution in the PCA plots is the aging time, which in both plots is positively correlated to the first component. As can be seen, this strong effect is due to the decreases of the levels of the labile odorants linalool and geraniol with the aging time, and to the continuous increase of the odorants derived from carotenoids or from phenols. The time effects are so strong that they partially shadow the effects of the yeast strains. These effects, however, are clearly perceptible, since in both cases there is a clear separation attending to the yeast strains carrying out the fermentation. Remarkably, the samples fermented with Sc are more distant to the unfermented controls than those previously inoculated with non-*Saccharomyces*, suggesting that the overall and average effect of non-*Saccharomyces* is to modulate or limit some of the changes induced by Sc on the varietal aroma.

The presentation of results will focus on some of the most relevant varietal aroma compounds for which significant effects of yeast were observed: TDN and vitispirane, linalool, geraniol and α-terpineol and vinyl phenols. For other relevant aroma compounds, either no relevant effects were noted (β-damascenone, guaiacol, isoeugenol, 2,6-dimethoxyphenol, vanillin and acetovanillone) or there were inconsistent trends because low levels were observed (in the cases of b-ionone and ethyl cinnamate) (see Tables S2–S5).

### 3.8. TDN and Vitispirane

**Figure 6:**
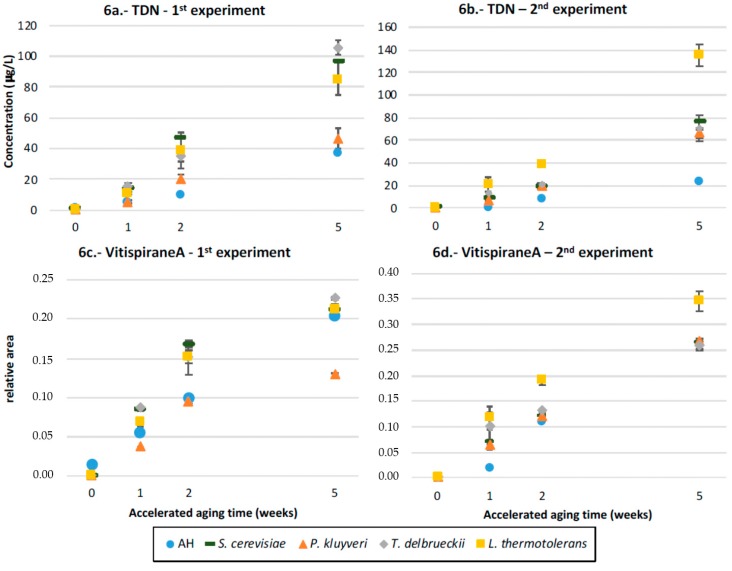
The norisoprenoids evolution in Riesling and Garnacha: the evolution of (**a**,**b**) TDN and (**c**,**d**) vitispirane A in wines fermented with different yeast strains or unfermented controls spiked with glycosidic precursors from Riesling (1st experiment) and Garnacha (2nd experiment) and its evolution during accelerated aging. The vitispirane samples are given in the relative area.

The results for these two aroma compounds are seen in Figure 6a–d. It can be observed that TDN is almost absent in recently fermented wines and increases with time at different rates, depending on the existence of fermentation and on the yeast strain that was first inoculated. Fermentation seems to be essential to form TDN since unfermented controls always had the lowest TDN levels. It can also be seen that the differences between yeasts are extraordinarily important; the low levels of TDN found in samples first inoculated with Pk are particularly interesting, in all cases producing the smallest levels of this aroma molecule. The highest levels of TDN produced by Lt in the batch fermented with the precursors extracted from Garnacha are also remarkable. In the case of vitispirane, whose levels also increase during aging from nearly null initial levels, the fermented samples have levels that are slightly higher than those of the unfermented controls, except for Pk in the first batch with precursors extracted from Riesling. The differential effects observed in TDN for Pk and Lt are also seen here, albeit with a smaller intensity.

**Figure 7:**
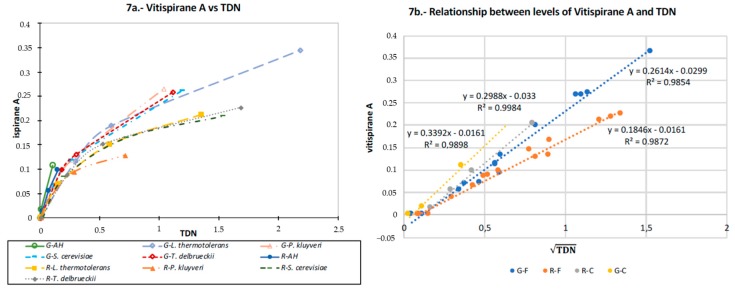
(**a**) TDN relative areas versus those of vitispirane A for all fermented samples and unfermented controls (AH) for the Garnacha (G) and Riesling (R) samples. The samples from the same condition (variety x yeast) aged at different times are represented with the same code and joined by a line. In all cases, the aged samples had increased levels of both compounds. (**b**) Levels of vitispirane A versus the square root of the TDN levels. The samples were classified into four categories: unfermented controls with Garnacha precursors (G-C); unfermented controls with Riesling precursors (R-C); fermented samples spiked with Garnacha precursors (G-F); and fermented samples with Riesling precursors (R-F). The plot demonstrates the existence of a linear relationship within each category.

The TDN and vitispirane levels increase at quite different rates with aging. This can be seen in the plots shown in Figure 7a, which relates both compounds. It can be observed that if the levels of vitispirane increase linearly with the aging time, the levels of TDN do so following a quadratic trend. Moreover, it was observed that the specific relationship between both compounds depends on the variety from which the precursor was extracted, and on the existence of fermentation. The yeast genera which starts fermentation seems to play a minor role (if any) in this mathematical relationship. In fact, all the samples fermented with Garnacha precursors follow very similar trends, while in the case of Riesling only the most aged sample fermented with Pk departs from the trend followed by all of the other samples. These results are further confirmed in the plot shown in Figure 7b, which represents the levels of vitispirane versus the square root of the levels of TDN. The samples have been grouped in four categories, related to the existence of fermentation and to the varietal origin of the precursors. The plot confirms the existence of a linear relationship between vitispirane and the square root of TDN, and the dependence of such a relationship on the varietal origin of the precursors and on the existence of fermentation. It can be also seen that the slopes follow the order:

Unfermented Garnacha Precursors > Unfermented Riesling Precursors > Fermented Garnacha > Fermented Riesling.

### 3.9. Terpenols

**Figure 8:**
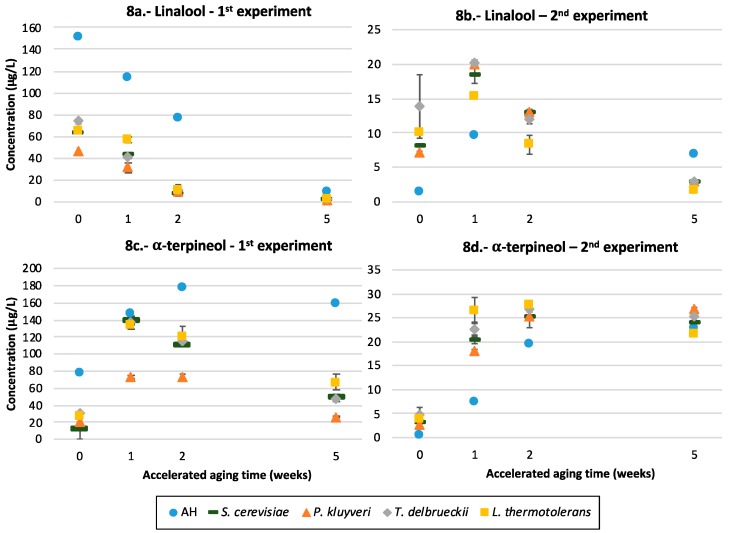
The linalool and α-terpineol formation in Riesling and Garnacha—the average concentration of (**a**,**b**) linalool and (**c**,**d**) α-terpineol in wines fermented with different yeast strains or unfermented controls spiked with glycosidic precursors from Riesling (1st experiment) and Garnacha (2nd experiment) and its evolution during accelerated aging.

Three aroma components are included in this group: linalool, geraniol and α-terpineol. The specific trends observed for linalool are observed in Figure 8a,b. The evolutions depend on the grape variety from which the precursors were extracted, and in fact the major differences are found between the unfermented controls of both varieties. In Riesling, which contains levels of linalool nearly one order of magnitude above those of Garnacha, maxima levels of linalool are obtained in the first time point, and the fermented samples do always show smaller levels of this aroma compound. In Garnacha, the highest levels are found after a short period of aging (1 week of AA), and the fermented samples do contain higher levels, except in the last point.

Regarding the differential effects played by yeasts, the differences between strains are not very intense. In the case of Riesling, Pk produced significantly smaller levels of linalool and geraniol in the recently fermented wine and after 1 week of AA, while the wine made with Lt had significantly higher levels of linalool and geraniol after 1 week of AA. In the case of Garnacha, Td induced highest levels of linalool and of geraniol in the young wine and after 1 week of AA, while the unaged wines made with Pk had the smallest levels. The evolution of α-terpineol, shown in Figure 8c,d, confirms that the wines made with Pk have the smallest levels of this compound.

### 3.10. Volatile Phenols

**Figure 9:**
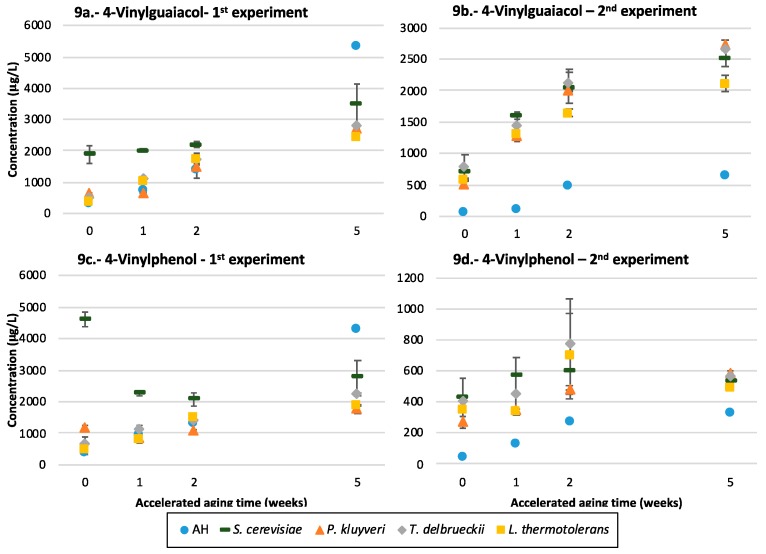
The volatile phenols formation in Riesling and Garnacha—the average concentration of (**a**,**b**) 4-vinylguaiacol and (**c**,**d**) 4-vinylphenol in wines fermented with different yeast strains or unfermented controls spiked with glycosidic precursors from Riesling (1st experiment) and Garnacha (2nd experiment) and its evolution during accelerated aging.

The two most relevant aroma compounds in this family are 4-vinylguaiacol (VG) and 4-vinylphenol (VP), shown in Figure 9. The first observation is that Riesling precursors have a much higher potential to form vinylphenols than those from Garnacha, which is well reflected in the levels of these compounds found in the aged unfermented controls: up to one order of magnitude higher in Riesling. Second, such a huge difference is not so clearly observed when comparing the fermenting samples of both varieties; i.e., the effects of fermentation in Garnacha outstands those observed for Riesling. Third, the samples fermented exclusively by Sc in Riesling follow a completely different pattern, with the highest levels of both compounds found in the unaged samples. Regarding the differential effects of yeasts, the differences between non-*Saccharomyces* are very little in the first experiment with the Riesling precursors (Figure 9a,c), except for the first time point, in which the samples made with Pk had higher levels than those of Td and Lt. In the experiment with the Garnacha precursors, the samples made with Lt had significantly smaller levels of VG (Figure 9b) and of VP after 1 week of AA (Figure 9d), while the samples fermented with Pk had levels of both compounds significantly smaller than those of Sc in the young wines and after 1 week of AA (also 2 weeks of AA for 4VP).

## 4. Discussion

### 4.1. Differential Fermentative Profiles

As observed in the relative positions in the PCA plots in Figure 2, the three non-*Saccharomyces* strains create or induce a differential and somehow complementary profile of fermentation volatiles that are linked to different aroma profiles.

One of the few common and sensory relevant features of non-*Saccharomyces* is the reduction of the levels of isoamyl alcohol. Decreased levels in this compound in mixed fermentations with Td [14], and particularly with Lt [7,24,25,26,27], have been already noted. This reduction will have sensory effects, since isoamyl alcohol exerts a strong aroma suppression effect on fruity, woody and other aroma nuances [15] and contributes to alcoholic (spirit-like) nuances [37].

### 4.2. P. kluyveri

The fermentative profile of Pk is additionally characterized by a systematically large production of the acetates of higher alcohols, and eventually, in a batch and precursor-dependent mode, by the production of higher levels of fatty acids and their ethyl esters (Figure 3c,e) as well as of branched acids and their ethyl esters (Figure 4a–d).

The fact that these positive interactions of Pk with Sc are batch-dependent suggests that they are strongly dependent on the nutrient profile and physiological state of the strains. These physiological effects will have to be well understood if the abilities of Pk to induce enhanced levels of fruity esters are to be successfully exploited. Similarly, the positive effect in the esterification ratios of fatty acid ethyl esters (Figure 3d,f) and the negative effect on the isobutyric acid and ethyl isobutyrate levels (Figure 4a,c) associated with the presence of the precursors fraction, may be linked to the likely presence of esterols in such fractions, which should be further investigated.

The high levels of higher alcohol acetates confirm previous reports about the ability of *Pichia* genera to form these compounds [4,5,6,38]. This production is going to have a strong sensory impact, since these compounds, particularly isoamyl acetate and phenylethyl acetate, are produced at levels at which they will become impact aroma compounds (their odor thresholds have been estimated to be 30 and 250 mg/L, respectively [39]). Since they are produced by Pk at levels that are much higher than those corresponding to the acetic acid + alcohol/acetate ester equilibria, they tend to hydrolyze with time, so that the levels are always maxima in young wines.

### 4.3. T. delbrueckii

The fermentative profile of Td is chiefly characterized by its increased levels of branched acids and their corresponding ethyl esters (Figure 4a–d). These esters are produced in fermentation at levels well below those corresponding to the esterification equilibria, so that they progressively increase during aging to become the most important source of fruitiness in aged red wines [39]. As a result, the differences of esters levels between yeast strains become more evident at longer aging times, which can explain some of the discrepancies found in the literature. The same results are observed for the other important esters in this group, ethyl 2-methylbutyrate and ethyl isovalerate, which suggests that these esters should be also specifically linked to the Td metabolism, as was demonstrated in the case of ethyl isobutyrate [17].

Regarding the other relevant fermentative aroma compounds, the positive effects exerted by Td on the levels of some acetates reported by other authors [17] are not observed here, while the previously observed [11] negative effects on the levels of fatty acids and their ethyl esters produced by Sc are confirmed (Figure 3c–f).

Considering the different evolution pattern with time of the different fruity esters and acetates, it can be concluded that Td will produce young wines with a reduced fruitiness (low acetates, and low fatty acids and ethyl esters of fatty acids), but aged wines with enhanced fruitiness (high contents in ethyl esters of branched acids). It can also be hypothesized that most of the positive sensory effects on red wine aroma attributed by different authors to this yeast [11,14,17] are specifically linked to its ability to produce high levels of branched acids and of their corresponding ethyl esters. The odor threshold for ethyl butyrate has been estimated to be 15 µg/L [39].

### 4.4. L. thermotolerans

Finally, the fermentative profile induced by Lt is mainly characterized by a strong reduction in fatty acids and their ethyl esters levels (Figure 3c–e) and by specific large increases in ethyl lactate and butanol. These last compounds, however, should not have a large sensory impact, because of their high odor thresholds [39]. From a sensory point of view, together with the known ability of Lt to increase acidity, this will lead to rather neutral and fresh wines in which other aroma notes, such as those derived from glycosidic precursors, can be easily perceived.

### 4.5. Effects on Varietal Aroma Profile

#### 4.5.1. TDN

TDN is a relevant aroma compound which for a long time has been known to be responsible for the kerosene-off odor developed by some wines during aging, particularly by those made with Riesling from warm areas [40,41,42]. Its threshold has been reported to be just 2 µg/L [42]. In recent years, a major concern about the increase in its levels, as a consequence of climate change, has been raised [43]. A first important observation is that Garnacha glycosidic precursors have a potential to form TDN that is equivalent to that of Riesling. To the best of our knowledge, this has not been previously observed, although in a previous report [44] precursors extracted from Garnacha and Shiraz were found to produce much higher levels of TDN (4–5 times higher) than other varieties, but Riesling was not among the studied varieties. Conversely, in a comparative study in which Riesling wines were found to have TDN levels that were on average 5 times higher than wines from other varieties, neither Garnacha or Shiraz were present among the studied samples [42].

**Figure 10:**
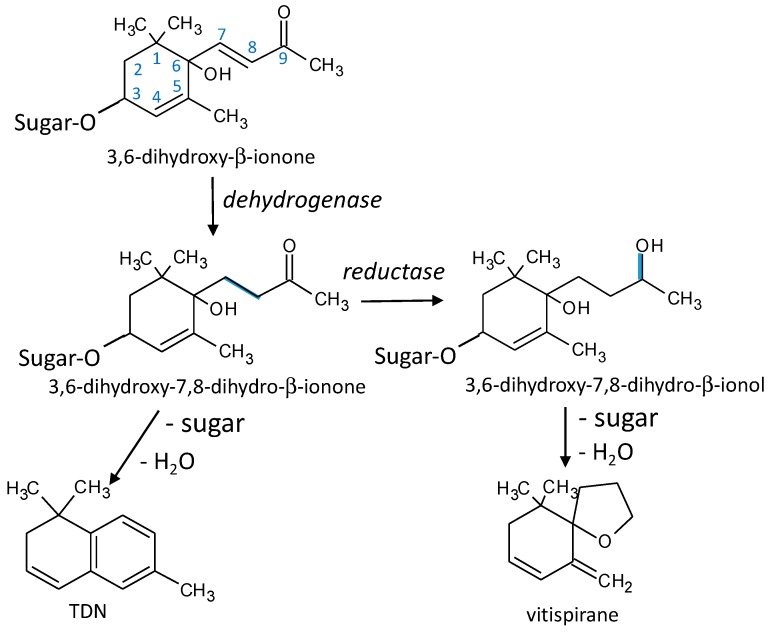
TDN and vitispirane formation from the glycosidic precursors: the formation of TDN and vitispirane from a common glycosidic precursor via enzymatic hydrolysis.

Overall, the results are consistent with our present understanding of the formation routes of TDN from different degradation products of carotenoids [41,43,45,46,47,48]. As schematized in Figure 10, the most relevant precursors in grapes are not well characterized glycosides of 3,6–dihydroxy–β-ionone and of its reduction product, 3,6–dihydroxy–7,8–dihydro–β–ionone [41]. This last compound can exist in four different tautomeric forms (not depicted in the scheme), all of which may also form glycosides. All these precursors can form TDN by a series of spontaneous, but slow, reactions at the wine pH; however, some of these reactions, such as the reduction of the double bond between C7 and C8, or the cleavage of the glycosidic bond, require (or are at least much accelerated by) the action of yeasts. The action of yeasts is also essential for reducing the keto function from the ionone to the OH function of the ionol, transforming the TDN precursor into the vitispirane precursor. These two precursors undergo the cleavage of the glucoside bond (enzymatically or by spontaneous acid hydrolysis) and further spontaneous dehydration reactions yielding vitispirane and TDN. In relation to the relationships seen in Figure 7, the overall apparent kinetics of the processes would follow a first order kinetics for vitispirane and a second-order one for TDN.

The fact that the combination of yeasts carrying out fermentation do not alter the relationships between TDN and vitispirane, as demonstrated in the plots shown in Figure 7, suggests that the reason why Pk provokes a reduction in the levels of both molecules is because it directly transforms the initial precursor into a different molecule that is no longer able to yield any of these molecules. It should be noted that Winterhalter et al. suggested a TDN levels reduction by using baker’s yeast [47], but such a reduction involved a higher formation of vitispirane. The results presented here suggest that Pk follows a different and more efficient route in reducing the TDN levels. Analogously, the fact that Lt produced higher levels of TDN from the Garnacha precursors without decreasing the vitispirane levels suggests either that Lt is particularly efficient at reducing the double bond from the ionone precursor, or that it is able to form TDN from a different precursor. It may be suggested that Lt and Pk could be advantageously used to understand the complicated chemistry of norisoprenoids.

#### 4.5.2. Terpenols

Linalool and geraniol (thresholds at 25 and 20 µg/L, respectively [39]) are highly relevant aroma compounds actively contributing to the floral and citrus character of many wines, notably Riesling. α-terpineol is a known by-product of the degradation of linalool and geraniol, has less sensory importance and is found most often at levels below its threshold. The differences shown in the evolution of unfermented controls in Riesling and Garnacha (Figure 8a,b) suggest that the precursors extracted from Riesling are mostly polyols, while those from Garnacha are glycosides. Polyols do not require the hydrolysis of a glycosidic bond, but just dehydration, to form the linalool. Such dehydration would be rapid at a wine pH, rendering linalool very quickly, which explains why maxima levels in the unfermented control of Riesling are observed in the first time point. The formation of linalool from a diol was demonstrated by Williams, et al. nearly 40 years ago [49]. The known instability of linalool at a wine pH, and the inexistence of a pool of glycosidic precursors able to release linalool by slow acid hydrolysis, would explain why maxima levels are found in the first time point in Riesling. Moreover, the inexistence of glycosides would explain why fermentation does not represent any advantage in this case. The smaller levels of linalool found in fermented samples, in comparison with the unfermented control, could result from the co-evaporation of this volatile molecule with the CO_2_ involved in fermentation. In the case of Garnacha, as the precursors are glycosides whose spontaneous acid hydrolysis takes a longer time, the fermented samples show increased levels because of the glucolytic and glycolytic activities of the yeast enzymes. In this case, the maximum levels are attained after a short aging (1 week of AA): the moment at which the spontaneous degradation rate of the compound equals the formation rate by spontaneous acid hydrolysis from the pool of glycosidic precursors. Given that part of such a pool of glycosidic precursors is depleted by yeasts, the levels of linalool remaining after a long aging period are higher in the unfermented controls.

The positive effects in the linalool and geraniol levels induced by Td are consistent with those found in cherry wines [16] and in Shiraz [33], and the poor effects of Lt and Pk are consistent with those reported in Riesling [7] and Muscat [28]. The smaller overall levels of terpenols, including α-terpineol, observed in Pk, together with the results from TDN, may suggest that this strain is able to metabolize terpenols and norisoprenoids.

#### 4.5.3. Vinylphenols

**Figure 11:**
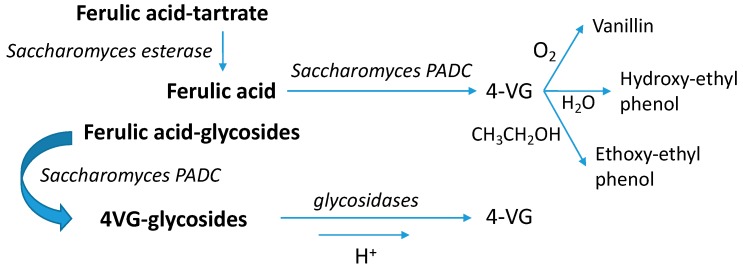
Scheme showing the main different processes leading to the production of 4-vinylguaiacol (4VG) during fermentation and aging.

4-vinylguaiacol and 4-vinylphenol have woody and roasted, and phenolic and medicinal aroma notes, respectively. With reported odor thresholds in wines of 40 and 180 µg/L, respectively [39], and with effective rejection thresholds at 725 µg/L for the 1:1 mixture [50], these compounds can be considered negative for the sensory characteristics of white wine at overly high levels [50]. Since these compounds can be produced by the decarboxylation of ferulic and coumaric acids, high levels are most often linked to yeasts with excessively high phenolic acid decarboxylase activities (PADC), which are most frequent in Sc [51,52]. Cinnamoyl esterase activities may also contribute by increasing the levels of free phenolic acids from the hydrolysis of their tartrate esters [53]. As PADC enzymes are inhibited by catechins [50], these compounds accumulate more in white wines. However, the phenolic acid precursors can also be present as glycosides [54], and even the odorants may also form glycosides [55]. Moreover, both odorants may not accumulate since they are reactive and can oxidize to form vanillins, hydrate to form hydroxyethyl derivatives [56], or react with ethanol to form ethoxyethyl phenols [53]. Figure 11 shows all these processes in a scheme.

With this at hand, the large differences between Riesling and Garnacha in the levels of volatiles produced by aging the unfermented controls (Figure 9a–d) suggest that Riesling precursors should be richer in glycosides of both odorants, since it is expected that glycosides are the single precursors producing sensible levels of vinylphenols by spontaneous acid hydrolysis. Second, the huge levels of vinylphenols observed in young samples containing Riesling precursors fermented with Sc (Figure 9a,c) suggest that the Riesling precursor fraction should contain much higher levels of free ferulic and coumaric acids, or eventually high levels of the tartrate esters, which is a known characteristic of Riesling wines [57]. Third, the fact that, in all cases, the levels of the phenols are smaller in the samples made with non-*Saccharomyces* suggests that these should not display strong decarboxylaxe activities. Fourth, the relatively large levels of odorants found in the aged fermented samples containing Garnacha precursors may suggest that the Garnacha precursor fraction should contain glycosides of the acids, which would be decarboxylated by yeasts, yielding glycosides of the vinylphenols.

In any case, the results suggest that all non-*Saccharomyces*, and particularly Lt, can, by an unknown interaction mechanism, strongly limit the fermentative production of vinylphenols by Sc, which should be of practical interest, since this would allow the use of Sc strains with high PADC activities without necessarily producing high levels of vinylphenols.

## 5. Conclusions

This study confirms and clarifies some previous results about the ability of non-*Saccharomyces* yeasts to modify the fermentative profile of wine aroma, introduces a time perspective and brings new results about the possibilities of modulating varietal aroma using a sequential fermentation approach. Regarding the fermentative aroma profile, the three non-*Saccharomyces* yeasts used in the study induced the formation of reduced levels of isoamyl alcohol. The pre-inoculation with Pk produced wines largely enriched in acetates of fusel alcohols, which was particularly noticeable in young wines. If these banana-pear-flowery aromas are targeted, Pk is the best choice. In some cases, Pk can also interact with Sc, inducing enhanced levels of fatty acids and of their ethyl esters, promoting additional fresh-fruity aromas. The pre-inoculation with Td markedly increased the levels of branched acids and their corresponding ethyl esters, but Td also interacts with Sc, inducing smaller levels of fatty acids and of their ethyl esters. As branched acid ethyl esters increase with aging, the positive effect of Td on fruity aroma will be more noticeable in aged wines, so that these species may be preferred for producing wines intended for aging. The pre-inoculation with Lt produced wines with less fatty acids and their ethyl esters and with more ethyl lactate, so that wines with a more neutral character will be obtained.

Regarding the varietal aroma compounds, the strongest effects of non-*Saccharomyces* yeasts are not related to increases in the levels of varietal aroma compounds, which are relatively modest, but to their ability to modulate the action of Sc on some relevant aroma precursors. The ability of Pk to induce smaller levels of TDN during aging via the likely metabolization of some of its precursors, or contrarily, the ability of Lt to produce higher levels of TDN and vitispirane from Garnacha precursors, are of particular importance. Pk seems to be a promising alternative for reducing the kerosene off-odor caused by TDN in wines from certain grapes, while Lt should be avoided in those cases. The abilities of the three strains to reduce the levels of vinylphenols produced by Sc in precursors extracted from Riesling, rich in phenolic acids, are also remarkable. In this regard, Lt and Pk may also induce significant reductions in the levels of vinylphenols in young wines. In the case of linalool and geraniol, the effects of sequential fermentation are only relevant if the precursor fraction is rich in glycosides. In these cases, Td and Lt can increase the contents of the varietal aroma in young, but not in aged wines.

## Figures and Tables

**Table 1:** The classical oenological parameters of synthetic wine: the characterization according to the classical oenological parameters of recently fermented wines from the (**A**) Riesling or (**B**) Garnacha set-up obtained by the fermentation of synthetic musts spiked with precursors (coded PR for Riesling or PG for Garnacha) or not (coded CTL). The data are the means of two biological replicates.

**(A) Riesling**	**Volatile Acidity**	**pH**	**Total Acidity**	**Residual Sugars**
CTL *S. cerevisiae*	0.51 ± 0.07	3.32 ± 0.06	5.06 ± 0.11	0.30 ± 0.1
PR *S. cerevisiae*	0.48 ± 0.04	3.36 ± 0.05	5.03 ± 0.08	0.30 ± 0.2
CTL *P. kluyveri*	0.49 ± 0.02	3.52 ± 0.03	4.76 ± 0.04	0.5 ± 0.2
PR *P. kluyveri*	0.40 ± 0.0	3.56 ± 0.04	4.95 ± 0.08	0.20 ± 0.1
CTL *L. thermotolerans*	0.22 ± 0.0	3.46 ± 0.02	4.65 ± 0.08	0.20 ± 0.1
PR *L. thermotolerans*	0.26 ± 0.0	3.49 ± 0.01	4.58 ± 0.0	0.05 ± 0.05
CTL *T. delbrueckii*	0.35 ± 0.02	3.52 ± 0.01	4.91 ± 0.11	0.15 ± 0.05
PR *T. delbrueckii*	0.24 ± 0.02	3.58 ± 0.01	4.80 ± 0.23	0.20 ± 0.0
**(B) Garnacha**	**Volatile Acidity**	**pH**	**Total Acidity**	**Residual Sugars**
CTL *S. cerevisiae*	0.6 ± 0.03	3.43 ± 0.01	6.5 ± 0.05	0.75 ± 0.07
PG *S. cerevisiae*	0.6 ± 0.08	3.43 ± 0.01	6.46 ± 0.11	1.75 ± 0.78
CTL *P. kluyveri*	0.9 ± 0.03	3.5 ± 0.08	6.27 ± 0.27	1.5 ± 0.71
PG *P. kluyveri*	0.93 ± 0.03	3.46 ± 0.02	6.16 ± 0	1 ± 0.14
CTL *L. thermotolerans*	0.79 ± 0	3.26 ± 0.01	8.89 ± 0.11	5.85 ± 1.41
PG *L. thermotolerans*	0.71 ± 0.03	3.44 ± 0.16	7.98 ± 0.32	11 ± 0.85
CTL *T. delbrueckii*	0.75 ± 0.03	3.41 ± 0.08	6.92 ± 0	4.5 ± 0.14
PG *T. delbrueckii*	0.75 ± 0.03	3.46 ± 0.01	6.57 ± 0.05	8 ± 0.71

## Supplementary Materials

The following are available online at https://www.mdpi.com/2076-2607/7/6/164/s1, Figure S1: Fermentation rate, Table S1: Mass spectra ions, Table S2: Volatiles quantified in Riesling experiment, Table S3: Volatiles quantified in Garnacha experiment, Table S4: ANOVA results for Riesling experiment, Table S5: ANOVA results for Garnacha experiment.
