# Supplementary material for: Modulating Fermentative, Varietal and Aging Aromas of Wine Using non-Saccharomyces Yeasts in a Sequential Inoculation Approach
Source: Microorganisms. 2019 Jun 6;7(6):164. doi: 10.3390/microorganisms7060164 (PMC6616922; doi:10.3390/microorganisms7060164)
Supplement: Supplementary file 1 [file microorganisms-07-00164-s001.pdf]

Supplementary Data

Table S1. Mass spectra ions selected to quantify minor and trace compounds using GC-MS.

Compounds	RT	*m/z*
**Ethyl esters and acetates**		
Ethyl isobutyrate	7.5	71[a], 116
Ethyl 2-methylbutyrate	12.0	57[a], 102
Ethyl 3-methylbutyrate	13.10	88[a], 115, 70
Ethyl 4-methylpentanoate	24.15	88[a], 101
Ethyl cyclohexanoate	45.55	83[a], 101, 156
Isobutyl acetate	9.71	56[a], 73
Phenylethyl acetate	79.90	91[a]
**Norisoprenoids**		
Rose oxide	39.73/ 40.93	139[a], 154
Vitispirane*	52.8/ 53.08	192[a], 93, 121, 171
Riesling acetal*	59.9	138[a], 125, 133
β-damascenone	79.86	69[a], 190
α-ionone	72,4	121[a], 93, 192
β-ionone	77.08	177[a], 192
1,1,6-Trimethyl-1,2-dihydronaphthalene (TDN)	66.48	157[a], 142, 172
**Monoterpenes**		
Linalool	55.01	71[a], 93, 121
α-terpineol	64.05	93[a], 121, 136
Geraniol	72.63	69[a], 123
β-citronellol	68.13	69[a], 81, 123
**Lactones**		
δ-nonalactone	81.81	85[a], 100
δ-decalactone	87.36	85[a], 100
Whiskylactone	74.56/ 78.18	99[a], 114
**Cinnamates**		
Ethyl dihydrocinnamate	74.54	178[a], 133
Ethyl cinnamate	86.80	131[a], 176
**Volatile phenols**		
Guaiacol	73.5	109[a], 124
o-cresol	81.16	108[a], 79
m-cresol	85.35	108[a], 79
4-ethylguaiacol	82.17	137[a], 152
Eugenol	88.73	164[a], 149
E-isoeugenol	96.83	164[a], 149
4-ethylphenol	89.33	107[a], 122
4-propylguaiacol	85.96	137[a], 166
4-vinylguaiacol	90.14	150[a], 135
4-vinylphenol	99.04	120[a], 91
2.6-dimethoxyphenol	93.27	154[a], 139
4-allyl-2,6-dimethoxyphenol	104.87	194[a], 119
**Vanillin derivates**		
Vanillin	105.85	151[a], 152, 123
Acetovanillone	108.83	166[a], 123
Syringaldehyde	127.15	182[a], 181, 167

[a] quantificatier ions.

Table S2. Average concentration of volatiles measured above the limit of quantification in control wines and those spiked with glycosidic precursors from Riesling grapes in unfermented controls (Acid Hydrolysis) and in wines fermented with S. *cerevisiae. P. kluyveri. T. delbrueckii and L. thermotolerans.*

Mosto	Control				PR				CTL	CTL	CTL	CTL	PR				CTL				PR			
Levadura	AH								*S. cerevisiae*								*P. kluyveri*							
Aging	0	1	2	5	0	1	2	5	0	1	2	5	0	1	2	5	0	1	2	5	0	1	2	5
Ethyl acetate	0	0	0	0	0	0	0	0	5625 ± 103	9936 ± 1171	13483 ± 1107	16193 ± 1708	30264 ± 262	29553 ± 1016	13523 ± 13523	23211 ± 778	28147 ± 996	29245 ± 131	27402 ± 1792	20699 ± 950	23011 ± 22656	22606 ± 21452	32724 ± 2401	24590 ± 592
Isoamyl acetate	0	0	0	0	0	0	0	0	30.6 ± 2.9	19.0 ± 3.8	14.8 ± 0.7	14.9 ± 4.5	32.4 ± 1.2	24.1 ± 0.0	17.1 ± 5.9	15.3 ± 1.0	492 ± 35	385 ± 38	416 ± 69	120 ± 19	395 ± 27	295 ± 65	283 ± 1	95.7 ± 13.9
Isobutyl acetate	1.75	1.50	1.18	1.26	2.39	1.60	1.79	1.39	17.2 ± 1.2	16.3 ± 1.1	16.9 ± 1.5	18.1 ± 1.2	20.5 ± 1.6	20.9 ± 0.3	20.7 ± 0.1	21.1 ± 1.0	263 ± 1	211 ± 3	165 ± 6	59.0 ± 5.0	194 ± 4	148 ± 12	128 ± 6	51.5 ± 2.4
Phenylethyl acetate	0.00	0.75	0.00	0.00	4.84	4.54	4.92	3.75	22.7 ± 1.1	17.1 ± 0.6	13.1 ± 0.6	9.4 ± 0.5	23.6 ± 0.6	18.7 ± 0.2	15.0 ± 0.2	11.4 ± 0.1	3727 ± 55	3078 ± 53	2477 ± 119	864 ± 55	2713 ± 62	2112 ± 215	1780 ± 16	671 ± 12
Ethyl hexanoate	0	0	0	0	0	0	0	0	70.1 ± 4.3	71.4 ± 31.0	65.1 ± 1.5	66.2 ± 12.0	94.7 ± 11.9	96.7 ± 10.1	86.8 ± 14.7	86.3 ± 3.9	123 ± 5	167 ± 14	129 ± 20	131 ± 3	162 ± 25	180 ± 62	171 ± 30	143 ± 28
Ethyl octanoate	0	0	0	0	0	0	0	0	104 ± 5	73.1 ± 30.8	99.3 ± 28.9	0.0 ± 0.0	138 ± 20	103 ± 4	111 ± 8	118 ± 10	168 ± 13	160 ± 17	189 ± 30	150 ± 1	146 ± 28	144 ± 13	148 ± 10	172 ± 33
Ethyl decanoate	0	0	0	0	0	0	0	0	34.2 ± 1.2	66.1 ± 11.4	54.3 ± 5.8	46.2 ± 27.5	47.9 ± 6.4	62.3 ± 13.6	48.0 ± 9.7	51.3 ± 3.4	87.9 ± 6.4	144 ± 47	92.1 ± 10.1	52.8 ± 4.7	141 ± 25	130 ± 83	73.8 ± 1.1	72.1 ± 17.7
Ethyl isobutyrate	0	0	0	0	0	0	0	0	1.1 ± 1.1	10.0 ± 0.5	21.9 ± 1.9	38.4 ± 6.1	1.4 ± 0.0	6.2 ± 0.4	13.6 ± 0.6	25.1 ± 2.7	4.2 ± 0.7	26.9 ± 1.6	48.8 ± 2.3	57.1 ± 1.2	2.7 ± 0.0	16.5 ± 0.8	33.3 ± 0.7	51.2 ± 17.0
Ethyl 2-methylbutyrate	0	0	0	0	0	0	0	0	0.0 ± 0.0	1.0 ± 0.1	2.3 ± 0.0	3.6 ± 0.5	0.6 ± 0.1	0.8 ± 0.0	2.0 ± 0.0	3.0 ± 0.5	0.0 ± 0.0	1.2 ± 0.1	2.4 ± 0.1	5.5 ± 0.1	0.6 ± 0.1	1.0 ± 0.1	2.5 ± 1.0	4.4 ± 0.9
Ethyl isovalerate	0	0	0	0	0	0	0	0	0.0 ± 0.0	0.9 ± 0.1	1.8 ± 0.2	3.4 ± 0.3	0.1 ± 0.0	0.7 ± 0.0	1.4 ± 0.1	2.8 ± 0.1	0.1 ± 0.0	0.9 ± 0.0	1.4 ± 0.1	3.9 ± 0.2	0.1 ± 0.0	0.6 ± 0.0	1.3 ± 0.1	3.1 ± 0.1
Ethyl lactate	0	0	0	0	0	0	0	0	100 ± 9	437 ± 9	668 ± 27	867 ± 68	110 ± 3	474 ± 18	841 ± 29	1158 ± 14	105 ± 24	413 ± 110	618 ± 136	1032 ± 311	117 ± 11	424 ± 19	785 ± 146	1226 ± 105
Isobutanol	0	0	0	0	0	0	0	0	8115 ± 946	7823 ± 1038	8226 ± 1900	7602 ± 966	8645 ± 441	8753 ± 201	9917 ± 197	9198 ± 159	8251 ± 510	9662 ± 714	8694 ± 186	7136 ± 552	9407 ± 3	9009 ± 1814	8744 ± 372	8409 ± 30
1-Butanol	0	0	0	0	0	0	0	0	60.0 ± 0.5	55.5 ± 4.0	55.2 ± 3.8	61.4 ± 2.4	63.8 ± 0.1	57.3 ± 6.6	58.4 ± 7.8	57.4 ± 8.8	47.1 ± 1.6	58.8 ± 13.3	63.6 ± 8.5	55.7 ± 0.7	54.0 ± 2.4	52.2 ± 0.7	53.8 ± 0.9	73.0 ± 18.4
Isoamyl alcohol	0	0	0	0	0	0	0	0	42922 ± 5794	41979 ± 6773	43341 ± 8181	41487 ± 5854	44279 ± 2897	44837 ± 1801	47300 ± 1647	45515 ± 2382	31823 ± 1978	33373 ± 3472	31011 ± 2881	31604 ± 2823	34994 ± 266	33848 ± 3496	35941 ± 1470	36035 ± 874
1-Hexanol	0	0	0	0	0	0	0	0	11.7 ± 0.1	7.5 ± 7.5	12.3 ± 0.1	9.9 ± 9.9	128 ± 4	137 ± 1	132 ± 3	141 ± 1	0.0 ± 0.0	0.0 ± 0.0	0.0 ± 0.0	5.4 ± 5.4	27.8 ± 0.4	31.3 ± 2.0	35.6 ± 0.5	39.1 ± 0.7
Metionol	0	0	0	0	0	0	0	0	2960 ± 112	3319 ± 360	3110 ± 439	3192 ± 220	3390 ± 166	3555 ± 64	3604 ± 38	3630 ± 107	3490 ± 75	4190 ± 251	4436 ± 217	4855 ± 23	3940 ± 29	4165 ± 860	5577 ± 733	5002 ± 90
β-Phenylethanol	0	0	0	0	0	0	0	0	4913 ± 521	5379 ± 717	5172 ± 788	5336 ± 643	5206 ± 139	5633 ± 257	5533 ± 40	5601 ± 215	5157 ± 53	5652 ± 391	6235 ± 352	7316 ± 42	5364 ± 188	5663 ± 580	7835 ± 983	7353 ± 46
γ-Butyrolactone	0	0	0	0	0	0	0	0	215 ± 41	932 ± 148	959 ± 98	965 ± 248	192 ± 8	870 ± 68	1025 ± 108	1050 ± 58	128 ± 86	875 ± 16	1021 ± 157	979 ± 50	240 ± 22	850 ± 99	1288 ± 233	1169 ± 124
γ-nonalactone	0.65	0	0	0.47	0	0	0	0	2.6 ± 1.6	4.7 ± 0.1	4.6 ± 0.0	4.9 ± 0.3	4.9 ± 0.1	4.9 ± 0.2	5.0 ± 0.1	5.3 ± 0.2	4.4 ± 0.1	5.0 ± 0.0	5.2 ± 0.1	5.2 ± 0.1	5.0 ± 0.3	5.0 ± 0.1	5.4 ± 0.4	5.5 ± 0.5
γ-decalactone	0	0	0	0	0	0	0	0	2.8 ± 0.4	2.9 ± 0.2	3.1 ± 0.1	3.0 ± 0.1	2.3 ± 0.1	2.8 ± 0.0	2.7 ± 0.2	2.9 ± 0.1	3.3 ± 0.3	3.9 ± 0.1	3.7 ± 0.2	4.2 ± 0.2	3.2 ± 0.1	3.7 ± 0.2	3.9 ± 0.3	4.3 ± 0.3
Butyric acid	0	0	0	0	0	0	0	0	285 ± 39	161 ± 27	104 ± 30	133 ± 42	360 ± 150	185 ± 15	210 ± 74	248 ± 3	2395 ± 2151	217 ± 6	442 ± 73	230 ± 82	278 ± 114	348 ± 186	262 ± 38	239 ± 12
Isobutyric acid	0	0	0	0	0	0	0	0	198 ± 16	219 ± 19	184 ± 25	233 ± 20	111 ± 2	98.7 ± 20.7	133 ± 5	141 ± 13	493 ± 24	500 ± 6	638 ± 54	332 ± 111	309 ± 9	272 ± 7	339 ± 1	294 ± 20
Hexanoic acid	0	0	0	0	0	0	0	0	1037 ± 113	1049 ± 63	1055 ± 15	1060 ± 78	1203 ± 61	1193 ± 18	1245 ± 58	1255 ± 43	2030 ± 48	2017 ± 179	2100 ± 146	2069 ± 9	2208 ± 134	2169 ± 222	2609 ± 37	2380 ± 51
Octanoic acid	0	0	0	0	0	0	0	0	2321 ± 122	2223 ± 200	2077 ± 97	2574 ± 69	2614 ± 95	2728 ± 33	2605 ± 155	2936 ± 113	3664 ± 374	3559 ± 229	3948 ± 460	4032 ± 134	3960 ± 214	3754 ± 358	4185 ± 171	4602 ± 213
Decanoic acid	0	0	0	0	0	0	0	0	960 ± 126	606 ± 63	610 ± 18	1067 ± 234	852 ± 149	741 ± 114	893 ± 105	1033 ± 17	1035 ± 131	796 ± 45	1271 ± 308	1417 ± 18	982 ± 58	1177 ± 414	1327 ± 5	1736 ± 226
TDN	0.84	0.27	0.27	0.20	1.61	5.06	10.4	37	1.5 ± 0.2	0.2 ± 0.0	0.3 ± 0.0	0.5 ± 0.5	1.4 ± 0.0	14.3 ± 0.7	47.4 ± 0.3	96.6 ± 0.4	0.5 ± 0.0	0.7 ± 0.0	0.0 ± 0.0	0.4 ± 0.0	0.5 ± 0.0	5.3 ± 0.8	20.1 ± 2.9	46.9 ± 6.4
β-damascenone	0	0	0	0	6.28	6.72	7.37	5.92	0.0 ± 0.0	0.1 ± 0.0	0.3 ± 0.0	0.3 ± 0.1	2.5 ± 0.0	3.4 ± 0.2	3.8 ± 0.0	4.5 ± 0.1	0.4 ± 0.0	0.5 ± 0.0	0.4 ± 0.0	0.3 ± 0.0	1.6 ± 0.0	2.6 ± 0.2	2.4 ± 0.2	3.0 ± 0.3
β-ionone	0.28	0.56	0.57	0.08	0.31	0.33	0.71	0.10	0.5 ± 0.0	0.2 ± 0.0	0.6 ± 0.1	0.5 ± 0.0	0.4 ± 0.0	0.2 ± 0.0	0.5 ± 0.0	0.4 ± 0.0	0.8 ± 0.0	0.3 ± 0.0	0.4 ± 0.0	0.1 ± 0.0	0.8 ± 0.1	0.3 ± 0.0	0.6 ± 0.2	0.3 ± 0.4
VitispiraneA[a]	0	0	0	0	0.01	0.06	0.10	0.20	0.00 ± 0.00	0.00 ± 0.00	0.00 ± 0.00	0.00 ± 0.00	0.00 ± 0.00	0.08 ± 0.00	0.17 ± 0.00	0.21 ± 0.01	0.00 ± 0.00	0.00 ± 0.00	0.00 ± 0.00	0.00 ± 0.00	0.00 ± 0.00	0.04 ± 0.00	0.09 ± 0.00	0.13 ± 0.00
RieslingAcetal[a]	0	0	0	0	0.06	0.18	0.28	0.33	0.00 ± 0.00	0.00 ± 0.00	0.00 ± 0.00	0.00 ± 0.00	0.00 ± 0.00	0.16 ± 0.02	0.18 ± 0.00	0.13 ± 0.01	0.00 ± 0.00	0.00 ± 0.00	0.00 ± 0.00	0.00 ± 0.00	0.00 ± 0.00	0.06 ± 0.06	0.13 ± 0.02	0.06 ± 0.01
Ethyl cinnamate	0	0.13	0.00	0.02	0.05	0.09	0.10	0.15	0.0 ± 0.0	0.0 ± 0.0	0.0 ± 0.0	0.0 ± 0.0	0.0 ± 0.0	0.0 ± 0.0	0.1 ± 0.0	0.1 ± 0.0	0.0 ± 0.0	0.0 ± 0.0	0.0 ± 0.0	0.0 ± 0.0	0.0 ± 0.0	0.0 ± 0.0	0.0 ± 0.0	0.0 ± 0.0
Linalool	0.76	2.10	1.98	0.29	150	114	76.7	9.37	4.2 ± 0.3	1.2 ± 0.1	2.8 ± 0.1	2.1 ± 0.1	62.9 ± 0.7	42.8 ± 1.3	7.1 ± 0.1	2.5 ± 0.0	2.2 ± 0.1	1.4 ± 0.1	1.9 ± 0.0	0.5 ± 0.0	46.3 ± 0.7	31.5 ± 4.9	9.4 ± 1.7	1.9 ± 1.4
α-terpineol	0.65	0.92	1.07	0.46	78.5	148	178	158	1.1 ± 0.1	1.5 ± 0.0	0.9 ± 0.5	1.0 ± 0.2	12.5 ± 12.0	139 ± 4	111 ± 1	50.1 ± 1.2	1.0 ± 0.0	0.9 ± 0.0	0.9 ± 0.1	0.3 ± 0.0	20.2 ± 0.4	73.1 ± 1.1	73.1 ± 2.7	26.1 ± 0.7
β-citronellol	0.23	0.71	0.63	0	0.43	0.60	0.86	0	2.3 ± 0.2	0.5 ± 0.0	0.8 ± 0.1	0.8 ± 0.2	6.9 ± 0.1	2.1 ± 0.0	1.1 ± 0.1	0.7 ± 0.1	1.3 ± 0.0	0.5 ± 0.0	0.6 ± 0.0	0.0 ± 0.0	4.5 ± 0.3	1.6 ± 0.1	1.1 ± 0.2	0.3 ± 0.5
Geraniol	0	1.75	0	0	38.7	36.2	26.6	1.88	2.6 ± 1.4	0.0 ± 0.0	0.4 ± 0.0	0.3 ± 0.3	9.6 ± 0.1	12.1 ± 0.0	1.7 ± 0.0	0.3 ± 0.3	0.4 ± 0.1	0.0 ± 0.0	0.5 ± 0.0	0.0 ± 0.0	5.5 ± 0.0	8.9 ± 1.6	51.2	38.5 ± 9.5
Guaiacol	0.06	0.04	0.06	0.00	0.41	0.38	0.58	1.86	0.3 ± 0.0	0.1 ± 0.0	0.1 ± 0.0	0.3 ± 0.1	2.2 ± 0.1	2.1 ± 0.1	0.9 ± 0.3	5.3 ± 3.8	0.2 ± 0.0	0.1 ± 0.0	0.1 ± 0.0	0.4 ± 0.1	0.9 ± 0.1	0.7 ± 0.1	1.0 ± 0.5	2.4 ± 0.6
4-vinylguaiacol	27.3	44.5	28.6	55.5	348	746	1404	5335	16.0 ± 9.9	27.1 ± 7.3	11.3 ± 4.4	67.0 ± 44.9	1902 ± 283	2016 ± 38	2207 ± 100	3496 ± 618	61.4 ± 2.1	24.0 ± 0.3	34.4 ± 8.1	106 ± 28	656 ± 11	682 ± 7	1506 ± 382	2705 ± 16
2-6-dimethoxyphenol	0.16	0	0	0.09	0	0	0	0	0.6 ± 0.2	0.1 ± 0.1	0.0 ± 0.0	0.5 ± 0.5	1.5 ± 1.5	0.0 ± 0.0	0.0 ± 0.0	4.1 ± 0.7	0.2 ± 0.2	0.0 ± 0.0	0.0 ± 0.0	0.0 ± 0.0	0.3 ± 0.0	0.0 ± 0.0	0.0 ± 0.0	1.4 ± 0.1
E-isoeugenol	0	0	0	0	0	0.19	0	0	0.0 ± 0.0	0.0 ± 0.0	0.0 ± 0.0	0.0 ± 0.0	0.4 ± 0.0	0.0 ± 0.0	0.2 ± 0.2	0.5 ± 0.0	0.0 ± 0.0	0.0 ± 0.0	0.0 ± 0.0	0.0 ± 0.0	0.0 ± 0.0	0.0 ± 0.0	0.0 ± 0.0	0.1 ± 0.1
4-vinylphenol	14.6	15.9	14	36.1	364	944	1318	4299	149 ± 23	26.0 ± 1.5	16.7 ± 3.6	54.0 ± 36.8	4599 ± 237	2255 ± 78	2079 ± 214	2791 ± 523	70.7 ± 0.5	18.4 ± 1.3	35.2 ± 13.6	124 ± 26	1165 ± 85	825 ± 9	1065 ± 51	1774 ± 153
vanillin	1.58	0.67	0	0.78	13.16	14.3	41.9	91	1.8 ± 0.7	2.5 ± 1.2	1.0 ± 0.0	3.7 ± 2.5	9.8 ± 1.6	9.5 ± 0.0	10.0 ± 0.8	23.5 ± 9.5	1.6 ± 0.2	0.9 ± 0.1	1.1 ± 0.1	1.8 ± 0.4	7.2 ± 0.4	7.4 ± 1.4	6.6 ± 0.3	8.7 ± 1.4
acetovanillone	0.76	0	0	0	5.02	5.75	8.30	13.9	0.0 ± 0.0	1.0 ± 0.7	0.0 ± 0.0	2.3 ± 1.8	58.4 ± 0.8	60.7 ± 0.1	58.8 ± 4.7	58.2 ± 2.2	1.5 ± 1.5	0.3 ± 0.3	0.2 ± 0.2	0.0 ± 0.0	32.9 ± 0.9	29.4 ± 1.4	30.8 ± 1.4	32.1 ± 1.7

[a] tentatively identified and given as relative area.

Table S2. (Cont.)

Mosto	CTL				PR				CTL				PR			
Levadura	*T. delbrueckii*								*L. thermotolerans*							
Aging	0	1	2	5	0	1	2	5	0	1	2	5	0	1	2	5
Ethyl acetate	15005 ± 370	14768 ± 1645	13958 ± 244	6059 ± 5986	43321 ± 1201	38412 ± 772	28832 ± 84	17938 ± 1208	13398 ± 2392	12579 ± 2117	11574 ± 1377	10963 ± 479	26460 ± 1251	733153 ± 3367	26992 ± 3771	17095 ± 3153
Isoamyl acetate	17.2 ± 2.2	16.9 ± 1.5	11.1 ± 1.4	0.0 ± 0.0	10.8 ± 1.9	11.0 ± 3.5	11.5 ± 1.9	0.0 ± 0.0	16.9 ± 2.4	12.3 ± 1.1	13.0 ± 3.3	0.0 ± 0.0	11.0 ± 1.4	16.3 ± 3.6	13.8 ± 2.0	12.5 ± 0.1
Isobutyl acetate	22.4 ± 0.2	21.2 ± 1.0	18.1 ± 0.9	15.6 ± 0.6	20.2 ± 5.4	18.0 ± 4.8	10.0 ± 1.6	13.8 ± 3.3	9.2 ± 0.4	8.0 ± 0.8	7.5 ± 0.3	7.5 ± 0.3	5.9 ± 1.8	6.4 ± 1.3	11.9 ± 5.5	7.5 ± 1.3
Phenylethyl acetate	48.9 ± 3.1	39.4 ± 3.0	32.7 ± 2.1	20.6 ± 1.1	48.0 ± 5.2	36.1 ± 4.0	16.6 ± 10.7	17.3 ± 1.2	3.6 ± 0.0	2.9 ± 0.1	2.6 ± 0.0	2.5 ± 0.1	5.3 ± 1.3	4.4 ± 0.9	18.7 ± 14.4	5.0 ± 0.5
Ethyl hexanoate	76.3 ± 6.1	67.1 ± 7.9	80.2 ± 7.6	59.6 ± 1.9	37.8 ± 21.0	39.7 ± 11.5	34.7 ± 5.9	0.0 ± 0.0	39.9 ± 1.5	37.9 ± 5.2	31.5 ± 2.4	32.3 ± 9.8	26.0 ± 5.6	43.7 ± 17.8	35.1 ± 6.0	21.5 ± 1.1
Ethyl octanoate	77.5 ± 10.3	66.7 ± 12.1	26.5 ± 26.5	0.0 ± 0.0	0.0 ± 0.0	27.5 ± 27.5	49.7 ± 19.8	0.0 ± 0.0	37.3 ± 6.1	27.0 ± 27.0	40.9 ± 4.7	34.7 ± 5.4	0.0 ± 0.0	20.9 ± 0.7	26.5 ± 9.4	25.1 ± 3.3
Ethyl decanoate	62.1 ± 0.2	53.8 ± 11.0	60.4 ± 1.5	74.8 ± 15.2	0.0 ± 0.0	48.0 ± 1.1	26.6 ± 1.7	0.0 ± 0.0	19.0 ± 19.0	43.2 ± 10.4	0.0 ± 0.0	27.5 ± 1.1	0.0 ± 0.0	0.0 ± 0.0	16.5 ± 1.1	0.0 ± 0.0
Ethyl isobutyrate	4.0 ± 0.3	25.7 ± 3.3	44.2 ± 2.0	93.2 ± 4.3	4.9 ± 1.2	27.0 ± 6.3	25.8 ± 9.8	77.9 ± 9.3	2.2 ± 0.4	5.2 ± 5.2	7.9 ± 7.9	31.2 ± 1.6	1.1 ± 1.1	6.4 ± 1.5	28.2 ± 17.9	26.6 ± 5.0
Ethyl 2-methylbutyrate	0.6 ± 0.0	1.7 ± 0.0	2.7 ± 0.2	5.9 ± 0.5	0.6 ± 0.0	1.8 ± 0.2	1.4 ± 0.5	5.2 ± 0.7	0.3 ± 0.3	0.3 ± 0.3	1.0 ± 0.2	1.1 ± 0.1	0.5 ± 0.0	0.4 ± 0.0	1.5 ± 0.8	2.3 ± 0.4
Ethyl isovalerate	0.0 ± 0.0	0.6 ± 0.0	1.1 ± 0.1	2.4 ± 0.2	0.0 ± 0.0	0.6 ± 0.1	0.6 ± 0.1	2.1 ± 0.5	0.0 ± 0.0	0.1 ± 0.1	0.2 ± 0.2	0.6 ± 0.1	0.0 ± 0.0	0.1 ± 0.1	0.7 ± 0.4	0.4 ± 0.4
Ethyl lactate	93.9 ± 10.8	335 ± 5	602 ± 49	1067 ± 184	119 ± 34	363 ± 93	588 ± 169	944 ± 176	364 ± 179	2313 ± 1097	3475 ± 1413	5141 ± 2459	1852 ± 1664	1060 ± 156	1839 ± 399	2910 ± 606
Isobutanol	7668 ± 225	7857 ± 293	8389 ± 482	8074 ± 654	7332 ± 1567	7865 ± 1237	7408 ± 1272	7344 ± 1344	5254 ± 632	5136 ± 244	5104 ± 197	5394 ± 277	4451 ± 452	4663 ± 962	4894 ± 917	4227 ± 675
1-Butanol	93.2 ± 1.8	105 ± 10	113 ± 2	110 ± 0	104 ± 6	102 ± 4	92.1 ± 13.5	93.5 ± 14.9	110 ± 2	115 ± 3	102 ± 16	96.9 ± 10.8	109 ± 1	110 ± 0	106 ± 4	91.3 ± 10.1
Isoamyl alcohol	35329 ± 1164	36262 ± 1071	37246 ± 957	39467 ± 1418	33266 ± 5029	34338 ± 4879	33867 ± 4951	34361 ± 4040	33012 ± 1798	33355 ± 102	32447 ± 744	32648 ± 730	34520 ± 5166	33537 ± 4542	35077 ± 5092	34306 ± 5380
1-Hexanol	12.1 ± 0.3	13.7 ± 0.8	14.0 ± 0.8	19.4 ± 2.0	130 ± 4	129 ± 1	137 ± 5	138 ± 3	12.0 ± 0.4	17.6 ± 2.1	14.4 ± 2.5	16.1 ± 0.5	159 ± 10	156 ± 2	156 ± 3	164 ± 5
Metionol	4114 ± 1	3871 ± 346	4315 ± 146	5400 ± 930	4355 ± 71	4220 ± 38	4446 ± 79	5587 ± 952	1483 ± 54	1616 ± 90	1307 ± 18	1616 ± 12	2404 ± 114	2188 ± 84	2291 ± 185	2389 ± 129
β-Phenylethanol	6538 ± 155	6240 ± 257	6571 ± 210	8521 ± 2431	6276 ± 304	5947 ± 347	6607 ± 431	7334 ± 677	3834 ± 148	4351 ± 22	4111 ± 133	4192 ± 320	4861 ± 911	4465 ± 567	4680 ± 938	4894 ± 879
γ-Butyrolactone	173 ± 6	657 ± 56	823 ± 31	1089 ± 204	237 ± 23	716 ± 38	811 ± 54	1076 ± 141	95.2 ± 49.5	959 ± 20	1022 ± 138	1115 ± 9	682 ± 503	819 ± 41	977 ± 65	1149 ± 36
γ-nonalactone	4.2 ± 0.0	4.2 ± 0.0	4.6 ± 0.0	4.5 ± 0.1	4.8 ± 0.1	4.8 ± 0.1	4.7 ± 0.5	5.3 ± 0.2	3.2 ± 0.1	3.1 ± 0.2	3.2 ± 0.2	3.6 ± 0.0	3.6 ± 0.3	3.7 ± 0.0	4.6 ± 0.8	4.2 ± 0.2
γ-decalactone	2.9 ± 0.0	2.9 ± 0.0	3.1 ± 0.1	3.2 ± 0.2	2.6 ± 0.1	2.8 ± 0.1	2.6 ± 0.2	3.0 ± 0.1	1.7 ± 0.2	2.1 ± 0.0	2.2 ± 0.0	1.9 ± 0.2	1.9 ± 0.1	2.1 ± 0.2	2.5 ± 0.6	2.1 ± 0.2
Butyric acid	102 ± 39	172 ± 3	89.0 ± 27.3	113 ± 63	116 ± 64	78.2 ± 23.3	168 ± 38	0.0 ± 0.0	494 ± 59	150 ± 21	356 ± 226	160 ± 59	229 ± 114	317 ± 225	242 ± 102	240 ± 104
Isobutyric acid	479 ± 13	479 ± 13	489 ± 11	413 ± 82	387 ± 59	410 ± 49	409 ± 26	446 ± 13	98.5 ± 44.8	166 ± 2	203 ± 37	227 ± 47	155 ± 6	144 ± 11	140 ± 22	143 ± 18
Hexanoic acid	1007 ± 59	948 ± 4	996 ± 8	1187 ± 263	603 ± 265	578 ± 256	650 ± 279	637 ± 249	488 ± 51	540 ± 54	342 ± 142	556 ± 12	301 ± 8	264 ± 1	291 ± 18	279 ± 14
Octanoic acid	1824 ± 31	1884 ± 207	1766 ± 172	1680 ± 386	1073 ± 513	1072 ± 535	1183 ± 481	1024 ± 502	984 ± 144	885 ± 55	895 ± 151	1002 ± 95	549 ± 116	505 ± 85	549 ± 60	602 ± 64
Decanoic acid	531 ± 36	500 ± 63	508 ± 98	559 ± 211	364 ± 178	1099 ± 929	509 ± 211	433 ± 197	628 ± 56	434 ± 32	762 ± 361	689 ± 73	814 ± 596	530 ± 318	559 ± 264	954 ± 457
TDN	0.5 ± 0.0	0.2 ± 0.0	0.7 ± 0.0	0.8 ± 0.3	0.5 ± 0.0	15.9 ± 1.2	35.2 ± 3.3	105 ± 5	1.3 ± 0.0	0.1 ± 0.1	0.1 ± 0.1	0.1 ± 0.1	0.5 ± 0.0	10.9 ± 0.7	38.8 ± 11.7	85.4 ± 10.5
β-damascenone	0.2 ± 0.1	0.3 ± 0.0	0.3 ± 0.0	0.1 ± 0.1	2.3 ± 0.3	3.1 ± 0.3	4.0 ± 0.5	4.4 ± 0.2	0.3 ± 0.0	0.1 ± 0.1	0.1 ± 0.1	0.2 ± 0.0	2.3 ± 0.3	3.2 ± 0.1	3.9 ± 0.2	4.0 ± 0.1
β-ionone	0.6 ± 0.0	0.5 ± 0.0	0.3 ± 0.0	0.6 ± 0.3	0.7 ± 0.0	0.5 ± 0.0	0.3 ± 0.0	0.4 ± 0.0	0.4 ± 0.0	0.3 ± 0.1	0.5 ± 0.0	0.4 ± 0.0	0.6 ± 0.0	0.2 ± 0.0	0.2 ± 0.0	0.3 ± 0.0
VitispiraneA[a]	0.00 ± 0.00	0.00 ± 0.00	0.00 ± 0.00	0.00 ± 0.00	0.00 ± 0.00	0.09 ± 0.00	0.15 ± 0.01	0.23 ± 0.00	0.00 ± 0.00	0.00 ± 0.00	0.00 ± 0.00	0.00 ± 0.00	0.00 ± 0.00	0.07 ± 0.01	0.15 ± 0.02	0.21 ± 0.00
RieslingAcetal[a]	0.00 ± 0.00	0.00 ± 0.00	0.00 ± 0.00	0.00 ± 0.00	0.00 ± 0.00	0.17 ± 0.00	0.27 ± 0.01	0.11 ± 0.01	0.00 ± 0.00	0.00 ± 0.00	0.00 ± 0.00	0.00 ± 0.00	0.00 ± 0.00	0.00 ± 0.00	0.22 ± 0.01	0.13 ± 0.01
Ethyl cinnamate	0.0 ± 0.0	0.0 ± 0.0	0.0 ± 0.0	0.0 ± 0.0	0.0 ± 0.0	0.1 ± 0.0	0.2 ± 0.0	0.2 ± 0.0	0.0 ± 0.0	0.0 ± 0.0	0.0 ± 0.0	0.0 ± 0.0	0.0 ± 0.0	0.0 ± 0.0	0.1 ± 0.0	0.2 ± 0.0
Linalool	2.4 ± 0.1	3.0 ± 0.0	1.5 ± 0.0	2.5 ± 1.2	73.8 ± 1.7	40.6 ± 1.1	10.2 ± 0.7	2.1 ± 0.0	3.5 ± 0.0	1.2 ± 0.1	1.7 ± 0.6	1.8 ± 0.1	65.3 ± 1.6	57.3 ± 2.5	11.2 ± 4.5	2.8 ± 0.5
α-terpineol	1.0 ± 0.0	1.6 ± 0.0	1.2 ± 0.0	1.0 ± 0.3	30.8 ± 1.9	138 ± 4	116 ± 3	48.4 ± 3.8	1.1 ± 0.0	1.2 ± 0.2	0.9 ± 0.3	0.7 ± 0.1	26.6 ± 1.6	134 ± 6	121 ± 11	66.8 ± 9.0
β-citronellol	1.2 ± 0.0	0.8 ± 0.0	0.5 ± 0.0	0.7 ± 0.3	5.6 ± 0.7	2.2 ± 0.2	0.7 ± 0.0	0.6 ± 0.1	1.4 ± 0.1	0.4 ± 0.1	0.3 ± 0.3	0.5 ± 0.1	4.4 ± 0.1	1.7 ± 0.2	0.8 ± 0.1	0.7 ± 0.1
Geraniol	1.1 ± 0.1	0.0 ± 0.0	0.3 ± 0.1	0.5 ± 0.6	13.3 ± 0.9	12.5 ± 1.4	2.9 ± 0.1	0.0 ± 0.0	1.1 ± 0.0	1.3 ± 1.0	1.4 ± 1.0	0.4 ± 0.1	12.4 ± 0.0	17.8 ± 1.1	3.5 ± 1.2	0.0 ± 0.0
Guaiacol	0.1 ± 0.1	0.1 ± 0.0	0.1 ± 0.0	0.2 ± 0.2	0.9 ± 0.3	0.4 ± 0.0	0.8 ± 0.0	1.1 ± 0.0	0.3 ± 0.1	0.1 ± 0.0	0.1 ± 0.0	0.1 ± 0.0	0.7 ± 0.1	0.7 ± 0.0	0.9 ± 0.3	0.9 ± 0.0
4-vinylguaiacol	17.6 ± 5.1	13.0 ± 6.0	23.0 ± 3.0	60.3 ± 35.7	525 ± 156	1114 ± 65	1719 ± 117	2827 ± 25	45.9 ± 15.0	13.9 ± 10.9	28.8 ± 24.8	22.2 ± 1.0	399 ± 58	1055 ± 29	1748 ± 186	2458 ± 56
2-6-dimethoxyphenol	0.9 ± 0.9	0.0 ± 0.0	0.1 ± 0.1	0.0 ± 0.0	0.0 ± 0.0	0.0 ± 0.0	0.0 ± 0.0	1.9 ± 1.9	0.0 ± 0.0	0.3 ± 0.1	0.2 ± 0.0	0.1 ± 0.1	0.0 ± 0.0	0.0 ± 0.0	0.0 ± 0.0	0.0 ± 0.0
E-isoeugenol	0.0 ± 0.0	0.0 ± 0.0	0.0 ± 0.0	0.0 ± 0.0	0.0 ± 0.0	0.0 ± 0.0	0.0 ± 0.0	0.5 ± 0.0	0.0 ± 0.0	0.0 ± 0.0	0.0 ± 0.0	0.0 ± 0.0	0.0 ± 0.0	0.0 ± 0.0	0.0 ± 0.0	0.0 ± 0.0
4-vinylphenol	22.1 ± 2.8	21.4 ± 6.5	15.8 ± 1.6	30.4 ± 23.8	643 ± 230	1094 ± 169	1383 ± 96	2243 ± 41	134 ± 71	75.7 ± 58.4	80.9 ± 57.4	10.2 ± 10.2	469 ± 126	793 ± 82	1463 ± 164	1874 ± 21
vanillin	8.1 ± 7.1	1.0 ± 0.1	0.9 ± 0.0	1.6 ± 0.5	5.5 ± 0.6	7.4 ± 0.5	7.4 ± 0.1	16.5 ± 10.0	1.1 ± 0.3	0.7 ± 0.4	0.4 ± 0.4	1.3 ± 0.1	5.4 ± 0.4	7.9 ± 0.5	8.2 ± 0.3	5.2 ± 0.2
acetovanillone	5.8 ± 5.8	0.4 ± 0.4	0.2 ± 0.2	0.6 ± 0.1	49.6 ± 4.5	43.6 ± 3.9	41.5 ± 2.3	36.6 ± 9.7	0.5 ± 0.5	0.0 ± 0.0	0.4 ± 0.4	0.0 ± 0.0	48.8 ± 3.5	47.2 ± 7.1	42.8 ± 3.2	27.8 ± 2.3

[a] tentatively identified and given as relative area.

Table S3. Average concentration of volatiles measured above the limit of quantification in control wines and those spiked with glycosidic precursors from Garnacha grapes in unfermented controls (Acid Hydrolysis) and in wines fermented with *S. cerevisiae. P. kluyveri. T. delbrueckii and L. thermotolerans.*

Precursors	Control				PG				Control				PG				Control				PG			
Yeast					AH				*S. cerevisiae*								*P. kluyveri*							
Aging	0	1	2	5	0	1	2	5	0	1	2	5	0	1	2	5	0	1	2	5	0	1	2	5
Ethyl acetate	0	0	0	0	159	81.4	113	89.8	47916 ± 25983	40643 ± 400	51373 ± 815	75038 ± 3552	98265 ± 815	103863 ± 1277	105766 ± 8826	111643 ± 2094	115396 ± 3465	53785 ± 53547	134323 ± 6716	126562 ± 6215	188802 ± 1965	181048 ± 5838	171344 ± 4821	154900 ± 482
Isoamyl acetate	0	0	0	0	0	0	0	0	226 ± 107	407 ± 18	325 ± 8	233 ± 5	377 ± 24	377 ± 1	319 ± 27	224 ± 2	3415 ± 147	2998 ± 332	2727 ± 279	1378 ± 17	2997 ± 128	2557 ± 10	2235 ± 100	1191 ± 60
Isobutyl acetate	0	0	0.66	0.63	7.3	4.2	6.1	5.3	94.6	88.6 ± 5.4	96.2 ± 7.0	87.6 ± 4.6	88.2 ± 0.3	83.1 ± 2.9	82.3 ± 0.7	80.6 ± 1.8	219 ± 12	195 ± 10	186 ± 5	171 ± 13	252 ± 5	205 ± 0	197 ± 3	176 ± 7
Phenylethyl acetate	0	0	0	0	0	0	0	0	133	96.9 ± 5.2	150 ± 67	54.5 ± 1.2	150 ± 3	103 ± 0	92.6 ± 4.3	59.9 ± 0.9		9454 ± 676	7253 ± 446	4765 ± 365	9850 ± 94	6837 ± 363	6056 ± 318	3570 ± 354
Ethyl hexanoate	0	0	0	0	0	0	0	0	49.5 ± 0.5	218 ± 20	192 ± 22	544 ± 4	0.0 ± 0.0	628 ± 40	602 ± 54	595 ± 35	165 ± 13	179 ± 12	236 ± 1	210 ± 39	359 ± 54	618 ± 120	535 ± 39	501 ± 2
Ethyl octanoate	0	0	0	0	0	0	0	0	123 ± 123	294 ± 124	369 ± 27	929 ± 13	382 ± 82	1038 ± 83	1046 ± 66	1028 ± 17	235 ± 38	198 ± 75	408 ± 44	308 ± 86	199 ± 37	694 ± 42	827 ± 47	793 ± 40
Ethyl decanoate	0	0	0	0	0	0	0	0	75.7 ± 75.7	104 ± 51	128 ± 11	234 ± 1	109 ± 9	493 ± 37	424 ± 40	128 ± 128	89.2 ± 11.4	50.6 ± 5.5	57.9 ± 57.9	77.5 ± 32.7	109 ± 13	265 ± 96	317 ± 15	155 ± 17
Ethyl isobutyrate	0	0	0	0	0	0	0	0	55.5	146 ± 9	250 ± 29	386 ± 6	30.8 ± 0.5	117 ± 10	157 ± 3	277 ± 0	35.0 ± 5.2	141 ± 3	216 ± 15	400 ± 44	40.2 ± 5.1	112 ± 11	177 ± 21	320 ± 7
Ethyl isovalerate	0	0	0	0	0	0	0	0	6.6	21.1 ± 1.5	34.5 ± 2.4	58.8 ± 4.1	4.7 ± 0.1	16.3 ± 1.1	23.8 ± 0.1	51.6 ± 3.4	4.4 ± 0.1	14.8 ± 0.3	26.0 ± 0.1	54.8 ± 4.5	3.6 ± 0.0	13.1 ± 0.8	20.3 ± 0.3	45.6 ± 1.1
Ethyl 2-methylbutyrate	0	0	0	0	0	0	0	0	5.0	14.9 ± 1.2	24.3 ± 0.1	43.4 ± 2.3	3.6 ± 0.0	11.8 ± 0.8	17.5 ± 0.4	37.5 ± 1.8	4.7 ± 0.3	15.2 ± 0.2	26.4 ± 0.7	53.3 ± 4.2	4.5 ± 0.9	11.1 ± 0.6	17.3 ± 0.6	40.2 ± 0.8
Ethyl lactate	0	0	0	0	0	0	0	0	597 ± 0	1973 ± 3	2929 ± 12	4638 ± 64	524 ± 20	2027 ± 58	2914 ± 42	5187 ± 84	724 ± 26	2895 ± 38	4954 ± 3	7909 ± 163	771 ± 12	3111 ± 55	4739 ± 220	8145 ± 156
Isobutanol	0	0	0	0	0	0	0	0	45325 ± 1531	43427 ± 6037	74678 ± 12438	41391 ± 4333	32287 ± 2788	33469 ± 1743	80738 ± 43781	34710 ± 62	50869 ± 6169	46562 ± 4653	72764 ± 21711	46414 ± 1564	45094 ± 1215	60932 ± 20067	86347 ± 11814	43866 ± 750
1-Butanol	0	0	0	0	0	0	0	0	282 ± 84	319 ± 28	568 ± 91	307 ± 36	361 ± 43	340 ± 8	811 ± 480	354 ± 34	205 ± 20	184 ± 1	290 ± 95	187 ± 14	186 ± 20	290 ± 102	401 ± 45	220 ± 3
Isoamyl alcohol	0	0	0	0	0	0	0	0	191149 ± 21601	213686 ± 16738	221999 ± 21432	220444 ± 17295	192907 ± 1604	187282 ± 1231	202726 ± 8881	197827 ± 7893	183988 ± 396	180723 ± 8648	188716 ± 4510	185446 ± 9139	182229 ± 2320	178591 ± 5591	184375 ± 10388	198478 ± 892
1-Hexanol	0	0	0	0	0	0	0	0	51.1 ± 2.2	31.0 ± 1.0	29.2 ± 3.6	27.0 ± 2.4	127 ± 14	101 ± 4	97.2 ± 2.4	99.6 ± 5.5	32.5 ± 0.6	28.5 ± 0.3	26.5 ± 0.3	24.9 ± 3.5	79.0 ± 10.3	55.0 ± 0.4	59.3 ± 2.0	57.4 ± 1.5
Metionol	0	0	0	0	0	0	0	0	6215 ± 63	6312 ± 500	7113 ± 223	7042 ± 105	6453 ± 209	7297 ± 18	7650 ± 703	6612 ± 209	9467 ± 187	9795 ± 574	11083 ± 103	11266 ± 208	10042 ± 207	11590 ± 696	11968 ± 402	12035 ± 82
β-Phenylethanol	0	0	0	0	0	0	0	0	43093 ± 6427	28107 ± 972	29620 ± 2488	32684 ± 5416	30153 ± 1324	33933 ± 112	36173 ± 4712	34833 ± 2229	30452 ± 1547	26911 ± 655	26205 ± 643	30113 ± 165	27534 ± 803	34197 ± 423	34468 ± 2013	30689 ± 422
γ-Butyrolactone	0	0	0	0	0	0	0	0	1128 ± 192	6520 ± 8	8341 ± 622	9626 ± 48	1384 ± 121	6994 ± 90	8923 ± 562	10098 ± 234	870 ± 38	4026 ± 76	5497 ± 197	6007 ± 123	972 ± 21	4820 ± 357	6143 ± 371	7403 ± 443
γ-nonalactone	0.79	0	0.67	0.56	0.97	0.95	1.43	1.54	7.6	8.1 ± 0.2	14.5 ± 6.9	7.6 ± 0.1	10.0 ± 0.3	9.1 ± 0.4	9.4 ± 0.3	9.0 ± 0.1	8.6 ± 0.1	7.7 ± 0.2	8.2 ± 0.1	8.3 ± 0.5	10.4 ± 0.1	10.0 ± 0.1	10.1 ± 0.1	9.9 ± 0.4
γ-decalactone	0	0	0	0	0	0	0	0	4.9	5.1 ± 0.0	9.5 ± 4.9	4.6 ± 0.1	5.1 ± 0.4	4.3 ± 0.1	4.1 ± 0.1	4.3 ± 0.0	6.7 ± 0.1	6.2 ± 0.2	6.2 ± 0.0	6.3 ± 0.4	6.8 ± 0.1	6.2 ± 0.0	6.0 ± 0.4	5.8 ± 0.0
Butyric acid	0	0	0	0	0	0	0	0	652 ± 107	520 ± 18	543 ± 70	504 ± 10	660 ± 26	541 ± 2	646 ± 52	675 ± 32	4020 ± 197	2777 ± 215	2718 ± 174	1887 ± 335	3421 ± 103	3180 ± 213	2767 ± 246	1476 ± 109
Isobutyric acid	0	0	0	0	0	0	0	0	4618 ± 2256	2467 ± 80	2467 ± 200	2548 ± 117	1831 ± 162	1796 ± 11	1902 ± 102	1905 ± 48	2732 ± 127	2480 ± 154	2588 ± 128	2701 ± 194	2113 ± 135	2145 ± 139	2232 ± 53	2032 ± 72
Hexanoic acid	0	0	0	0	0	0	0	0	1257 ± 826	1359 ± 5	1413 ± 86	1044 ± 40	1905 ± 83	1139 ± 26	1227 ± 65	1140 ± 28	1874 ± 31	1382 ± 109	1304 ± 155	1443 ± 170	1652 ± 6	1048 ± 39	1068 ± 51	1042 ± 12
Octanoic acid	0	0	0	0	0	0	0	0	3121 ± 2071	3658 ± 68	4202 ± 174	3590 ± 26	5384 ± 158	3851 ± 198	4451 ± 643	4107 ± 18	4965 ± 71	4112 ± 152	3942 ± 438	4264 ± 470	4853 ± 176	3847 ± 46	3946 ± 13	3235 ± 16
Decanoic acid	0	0	0	0	0	0	0	0	333 ± 333	752 ± 304	961 ± 82	1191 ± 12	727 ± 188	961 ± 237	991 ± 13	1349 ± 157	676 ± 12	479 ± 1	1146 ± 135	1087 ± 216	655 ± 8	632 ± 130	825 ± 10	908 ± 76
TDN	0	0	0	0	0	0.81	7.5	24	0.5	0.4 ± 0.0	0.0 ± 0.0	0.0 ± 0.0	0.8 ± 0.0	8.4 ± 0.5	18.8 ± 1.6	75.9 ± 6.5	0.7 ± 0.1	0.3 ± 0.0	0.0 ± 0.0	0.0 ± 0.0	0.8 ± 0.1	7.1 ± 1.6	19.1 ± 2.1	66.3 ± 4.9
β-damascenone	0	0	0	0	1	3.5	7.1	7.1	0.4	0.2 ± 0.0	0.1 ± 0.1	0.0 ± 0.0	2.1 ± 0.1	3.4 ± 0.1	3.9 ± 0.4	4.5 ± 0.3		0.2 ± 0.0	0.1 ± 0.1	0.0 ± 0.0	2.2 ± 0.1	4.1 ± 0.0	1.4 ± 1.4	4.1 ± 0.1
β-ionone	0	0	0	0	0	0	0	0	0.6	0.6 ± 0.1	0.3 ± 0.3	0.2 ± 0.0	0.5 ± 0.0	0.7 ± 0.1	0.2 ± 0.1	0.2 ± 0.0	0.8 ± 0.0	0.3 ± 0.0	0.5 ± 0.3	0.4 ± 0.3	0.5 ± 0.0	0.6 ± 0.2	0.5 ± 0.2	0.1 ± 0.0
VitispiraneA[a]	0	0	0	0	0	0.02	0.11	1.84	0.00 ± 0.00	0.00 ± 0.00	0.00 ± 0.00	0.00 ± 0.00	0.00 ± 0.00	0.07 ± 0.00	0.12 ± 0.01	0.26 ± 0.01	0.00 ± 0.00	0.00 ± 0.00	0.00 ± 0.00	0.00 ± 0.00	0.00 ± 0.00	0.06 ± 0.01	0.12 ± 0.01	0.27 ± 0.00
RieslingAcetal[a]	0	0	0	0	0	0.08	0.30	0.44	0.00 ± 0.00	0.00 ± 0.00	0.00 ± 0.00	0.00 ± 0.00	0.00 ± 0.00	0.18 ± 0.01	0.27 ± 0.01	0.39 ± 0.00	0.00 ± 0.00	0.00 ± 0.00	0.00 ± 0.00	0.00 ± 0.00	0.00 ± 0.00	0.16 ± 0.02	0.28 ± 0.02	0.40 ± 0.01
Ethyl cinnamate	0	0	0	0	0	0	0	0	0.0	0.0 ± 0.0	8.6 ± 8.6	1.1 ± 0.1	0.0 ± 0.0	0.2 ± 0.0	0.6 ± 0.1	1.6 ± 0.2	0.0 ± 0.0	0.1 ± 0.0	0.5 ± 0.2	0.0 ± 0.0	0.2 ± 0.0	0.4 ± 0.0	0.7 ± 0.4	1.0 ± 0.4
Linalool	0	0	0	0	1.5	9.6	12.7	6.9	3.3	2.3 ± 0.1	1.0 ± 0.2	0.8 ± 0.0	8.2 ± 0.0	18.4 ± 1.2	12.8 ± 0.1	2.8 ± 0.1	2.4 ± 0.1	2.0 ± 0.1	1.7 ± 0.3	0.8 ± 0.5	7.1 ± 0.1	19.9 ± 0.2	13.1 ± 0.3	2.9 ± 0.1
α-terpineol	0	0	0	0	0	7.4	19.5	23.0	1.1	2.1 ± 0.1	2.4 ± 0.1	1.6 ± 0.2	3.0 ± 0.0	20.5 ± 0.8	25.1 ± 2.0	23.8 ± 0.5	0.7 ± 0.1	1.5 ± 0.0	2.0 ± 0.1	1.8 ± 0.1	2.7 ± 0.1	18.1 ± 0.3	25.2 ± 0.3	26.7 ± 0.3
β-citronellol	0	0	0	0	0	0	0	0	2.6	1.8 ± 0.1	1.1 ± 0.1	0.3 ± 0.0	4.9 ± 0.2	3.5 ± 0.2	2.6 ± 0.1	0.9 ± 0.1	2.2 ± 0.2	1.4 ± 0.1	1.1 ± 0.1	0.4 ± 0.2	3.4 ± 0.1	2.6 ± 0.1	1.9 ± 0.2	0.8 ± 0.0
Geraniol	1	0	1	1	2	3	5	3	1.8	0.9 ± 0.0	0.0 ± 0.0	0.0 ± 0.0	3.5 ± 0.2	5.2 ± 0.3	3.6 ± 0.2	1.7 ± 0.0	2.5 ± 0.0	1.3 ± 0.1	1.1 ± 0.0	0.8 ± 0.8	4.4 ± 0.2	5.8 ± 0.2	4.2 ± 0.4	2.3 ± 0.2
Guaiacol	0	0	0	0	0	1	1	2	0.2	0.2 ± 0.0	0.2 ± 0.0	0.1 ± 0.0	4.3 ± 0.3	4.2 ± 0.1	5.1 ± 0.9	5.8 ± 0.6	0.1 ± 0.0	0.2 ± 0.0	0.2 ± 0.0	0.3 ± 0.1	3.2 ± 0.5	2.4 ± 0.2	4.0 ± 0.3	6.3 ± 0.5
4-vinylguaiacol	12.7	5.6	5.8	16.5	77.6	127	481	649	20.2	16.3 ± 3.1	18.8 ± 0.6	6.2 ± 1.3	720 ± 5	1588 ± 49	2045 ± 236	2516 ± 123	5.7 ± 1.3	13.7 ± 1.8	12.7 ± 4.3	21.9 ± 0.7	512 ± 59	1277 ± 87	1999 ± 5	2731 ± 75
2-6-dimethoxyphenol	2	0	0	0	0	0.9	3.1	7.0	0.2	0.3 ± 0.1	0.5 ± 0.3	1.0 ± 0.8	11.2 ± 2.6	13.8 ± 3.7	18.4 ± 7.7	19.8 ± 1.7	0.1 ± 0.0	0.2 ± 0.0	0.3 ± 0.1	0.8 ± 0.3	8.3 ± 0.9	4.9 ± 1.0	9.9 ± 0.7	20.2 ± 3.4
E-isoeugenol	0	0	0	0	0	0	0.6	0.7	0.0	0.0 ± 0.0	0.0 ± 0.0	0.0 ± 0.0	2.5 ± 0.0	2.8 ± 0.3	2.7 ± 0.4	2.4 ± 0.0	0.0 ± 0.0	0.0 ± 0.0	0.0 ± 0.0	0.0 ± 0.0	2.3 ± 0.3	2.2 ± 0.2	2.8 ± 0.0	2.8 ± 0.1
4-vinylphenol	9.7	4.1	5.3	8.9	38.6	123	264	324	21.5	9.7 ± 9.7	19.9 ± 0.7	9.3 ± 1.8	424 ± 5	569 ± 116	598 ± 174	525 ± 5	11.6 ± 2.7	17.8 ± 1.3	18.3 ± 4.6	22.0 ± 0.1	270 ± 36	346 ± 10	477 ± 24	578 ± 26
Vanillin	10.2	0.6	2.0	1.7	6.9	8.9	21.2	36.8	1.5	0.7 ± 0.1	2.3 ± 1.0	5.7 ± 3.7	10.8 ± 0.7	36.6 ± 23.8	21.5 ± 2.4	20.5 ± 1.2	1.0 ± 0.2	0.8 ± 0.1	1.3 ± 0.4	2.6 ± 1.5	9.4 ± 0.5	9.6 ± 0.2	13.9 ± 4.2	21.5 ± 0.6
Acetovanillone	7.5	1.2	1.1	0.9	53.2	4.2	6.4	12.9	2.5	1.2 ± 0.3	1.5 ± 0.8	3.3 ± 3.3	126 ± 45	194 ± 17	186 ± 6	166 ± 5	0.7 ± 0.1	1.4 ± 0.7	1.0 ± 0.3	2.6 ± 0.5	170 ± 10	131 ± 16	145 ± 34	185 ± 4

[a] tentatively identified and given as relative area.

Table S3. (Cont.)

Precursors	Control				PG				Control				PG			
Yeast	*T. delbrueckii*								*L. thermotolerans*							
Aging	0	1	2	5	0	1	2	5	0	1	2	5	0	1	2	5
Ethyl acetate	47032 ± 24509	75440 ± 1213	91377 ± 61	92847 ± 577	166213 ± 2999	155637 ± 7184	148887 ± 15598	127574 ± 458	59220 ± 56	66765 ± 299	67184 ± 1116	75513 ± 265	140554 ± 504	135488 ± 307	131983 ± 1955	100561 ± 177
Isoamyl acetate	295 ± 182	132 ± 1	122 ± 1	109 ± 8	111 ± 18	144 ± 0	137 ± 3	133 ± 2	217 ± 13	181 ± 7	150 ± 0	113 ± 4	233 ± 33	207 ± 0	167 ± 9	1901 ± 1784
Isobutyl acetate	88.6 ± 1.6	90.5 ± 1.4	87.6 ± 0.3	111 ± 6	140 ± 45	92.5 ± 0.2	98.2 ± 7.8	118 ± 4	66.4 ± 0.6	61.5 ± 2.6	62.2 ± 3.2	57.9 ± 3.2	67.4 ± 0.4	59.9 ± 3.7	60.4 ± 6.9	68.4 ± 5.6
Phenylethyl acetate	320 ± 9	327 ± 83	188 ± 1	142 ± 3	398 ± 139	351 ± 158	168 ± 5	116 ± 5	57.0 ± 7.3	41.2 ± 2.2	37.3 ± 2.5	36.0 ± 11.9	48.2 ± 0.3	57.0 ± 17.7	34.8 ± 1.7	32.4 ± 0.7
Ethyl hexanoate	115 ± 115	95.5 ± 6.5	148 ± 25	103 ± 2	0.0 ± 0.0	135 ± 19	137 ± 41	107 ± 24	48.2 ± 4.7	55.6 ± 1.1	25.2 ± 25.2	54.2 ± 0.2	57.2 ± 57.2	159 ± 2	152 ± 33	144 ± 30
Ethyl octanoate	268 ± 210	167 ± 7	167 ± 19	177 ± 21	63.8 ± 16.8	154 ± 4	157 ± 33	135 ± 1	93.1 ± 53.2	51.2 ± 8.0	51.4 ± 5.4	63.6 ± 8.1	180 ± 132	181 ± 18	134 ± 6	193 ± 45
Ethyl decanoate	0.0 ± 0.0	0.0 ± 0.0	0.0 ± 0.0	0.0 ± 0.0	0.0 ± 0.0	0.0 ± 0.0	0.0 ± 0.0	0.0 ± 0.0	49.5 ± 4.9	30.8 ± 3.4	34.2 ± 0.6	61.9 ± 38.1	59.3 ± 14.5	137 ± 40	54.8 ± 54.8	118 ± 27
Ethyl isobutyrate	114 ± 4	357 ± 15	505 ± 24	1011 ± 149	132 ± 36	296 ± 0	435 ± 4	816 ± 4	47.9 ± 1.6	139 ± 11	175 ± 36	327 ± 21	38.9 ± 2.1	78.6 ± 64.1	154 ± 19	371 ± 88
Ethyl isovalerate	2.2 ± 2.2	10.7 ± 0.4	16.8 ± 0.9	34.8 ± 2.6	5.2 ± 1.6	9.9 ± 0.1	15.6 ± 0.2	32.9 ± 1.4	2.3 ± 0.2	8.1 ± 0.0	13.2 ± 0.0	26.7 ± 1.8	1.7 ± 0.0	7.2 ± 0.2	11.8 ± 0.7	27.4 ± 0.2
Ethyl 2-methylbutyrate	4.6 ± 0.0	14.1 ± 0.8	21.8 ± 1.1	45.6 ± 2.9	5.7 ± 1.6	12.3 ± 0.4	20.7 ± 0.1	41.6 ± 0.9	2.9 ± 0.2	8.5 ± 0.4	14.2 ± 0.0	28.8 ± 0.7	2.1 ± 0.0	9.2 ± 0.1	12.7 ± 0.2	28.6 ± 0.8
Ethyl lactate	545 ± 54	2442 ± 21	3745 ± 28	6199 ± 11	662 ± 74	2531 ± 94	3882 ± 141	6348 ± 83	16111 ± 817	75862 ± 685	115387 ± 5327	186688 ± 7348	11845 ± 769	63983 ± 1630	95974 ± 2571	146020 ± 9999
Isobutanol	46525 ± 2868	75502 ± 20250	42812 ± 1791	45437 ± 1610	47816 ± 456	45798 ± 1892	44777 ± 335	50035 ± 4681	28429 ± 918	46129 ± 19150	59208 ± 32857	45903 ± 20089	24362 ± 3697	28357 ± 3159	24301 ± 1321	28164 ± 1491
1-Butanol	314 ± 61	391 ± 108	200 ± 5	219 ± 16	300 ± 5	270 ± 8	275 ± 26	319 ± 27	400 ± 9	643 ± 245	862 ± 474	700 ± 299	436 ± 1	448 ± 90	389 ± 3	474 ± 41
Isoamyl alcohol	199904 ± 33337	167528 ± 3780	169586 ± 8688	165996 ± 3231	165229 ± 782	153452 ± 2168	160373 ± 1153	159103 ± 1830	152611 ± 307	149919 ± 4033	156226 ± 7191	152385 ± 2096	145238 ± 6798	140945 ± 8614	147328 ± 9539	146940 ± 3959
1-Hexanol	49.7 ± 15.2	49.0 ± 0.8	49.1 ± 2.6	47.8 ± 4.4	149 ± 27	107 ± 10	108 ± 0	110 ± 2	33.3 ± 0.4	40.0 ± 3.8	52.8 ± 11.5	38.0 ± 10.7	133 ± 1	113 ± 1	120 ± 11	131 ± 3
Metionol	6110 ± 9	8165 ± 167	8098 ± 203	8572 ± 224	6363 ± 4	8104 ± 292	9006 ± 325	8776 ± 48	4911 ± 411	6167 ± 11	6519 ± 67	5753 ± 56	5324 ± 485	5890 ± 394	6072 ± 49	6112 ± 39
β-Phenylethanol	39683 ± 6635	57655 ± 5942	52489 ± 1223	59920 ± 6367	35655 ± 1161	45883 ± 2002	47021 ± 1223	45122 ± 1393	28106 ± 1776	25719 ± 68	27636 ± 789	22502 ± 1023	26220 ± 566	31365 ± 1782	30121 ± 2571	30003 ± 393
γ-Butyrolactone	1061 ± 110	3325 ± 133	3987 ± 169	4650 ± 311	919 ± 37	3213 ± 79	4194 ± 144	4543 ± 66	1548 ± 14	5418 ± 513	6681 ± 371	6665 ± 12	1626 ± 40	5445 ± 307	6666 ± 25	7160 ± 127
γ-nonalactone	9.8 ± 0.2	13.1 ± 4.9	8.6 ± 0.3	8.3 ± 0.3	17.7 ± 6.0	20.9 ± 10.8	10.2 ± 0.3	10.4 ± 0.5	8.1 ± 0.1	7.1 ± 0.1	6.7 ± 0.2	9.9 ± 3.1	9.1 ± 0.0	13.5 ± 5.2	7.7 ± 0.2	8.2 ± 0.1
γ-decalactone	8.4 ± 0.3	10.9 ± 3.9	7.0 ± 0.1	7.1 ± 0.3	11.5 ± 3.8	12.7 ± 6.2	6.2 ± 0.1	6.7 ± 0.3	33.9 ± 3.0	29.4 ± 0.4	28.2 ± 0.3	38.1 ± 11.2	31.7 ± 0.2	44.1 ± 17.7	26.4 ± 0.3	27.8 ± 0.1
Butyric acid	639 ± 73	397 ± 32	362 ± 35	494 ± 95	540 ± 29	426 ± 68	499 ± 52	445 ± 54	373 ± 12	329 ± 13	381 ± 54	269 ± 15	423 ± 100	275 ± 22	339 ± 24	289 ± 2
Isobutyric acid	4645 ± 1870	7041 ± 179	6593 ± 288	7413 ± 767	6339 ± 283	5603 ± 340	6046 ± 374	5682 ± 189	2012 ± 55	2195 ± 92	2466 ± 35	2765 ± 193	2098 ± 174	2048 ± 137	2137 ± 221	2298 ± 225
Hexanoic acid	1110 ± 698	263 ± 2	255 ± 6	231 ± 0	395 ± 26	262 ± 7	258 ± 13	253 ± 8	498 ± 43	409 ± 2	407 ± 12	330 ± 26	509 ± 57	274 ± 30	295 ± 31	279 ± 8
Octanoic acid	2696 ± 1634	922 ± 37	732 ± 7	871 ± 4	888 ± 120	728 ± 123	731 ± 10	672 ± 26	1263 ± 134	1138 ± 35	1093 ± 64	909 ± 112	1227 ± 80	886 ± 56	827 ± 29	12884 ± 12019
Decanoic acid	419 ± 371	96.2 ± 1.5	98.3 ± 2.3	151 ± 11	19.9 ± 19.9	105 ± 0	110 ± 0	161 ± 16	505 ± 31	585 ± 99	563 ± 26	736 ± 120	1096 ± 115	1316 ± 393	824 ± 39	1254 ± 318
TDN	0.0 ± 0.0	0.0 ± 0.0	0.0 ± 0.0	0.0 ± 0.0	0.1 ± 0.1	12.8 ± 5.3	21.4 ± 0.9	70.7 ± 10.9	0.1 ± 0.1	0.0 ± 0.0	0.0 ± 0.0	0.0 ± 0.0	0.2 ± 0.0	20.7 ± 6.2	39.3 ± 0.4	136 ± 9
β-damascenone	0.2 ± 0.0	0.0 ± 0.0	0.0 ± 0.0	0.0 ± 0.0	0.9 ± 0.6	4.6 ± 2.1	2.9 ± 0.2	3.7 ± 0.1	0.3 ± 0.1	0.0 ± 0.0	0.0 ± 0.0	0.0 ± 0.0	2.1 ± 0.3	4.7 ± 1.4	3.9 ± 0.4	4.6 ± 0.1
β-ionone	0.6 ± 0.0	0.2 ± 0.1	0.2 ± 0.0	0.1 ± 0.1	0.8 ± 0.2	0.3 ± 0.3	0.2 ± 0.0	0.1 ± 0.0	0.8 ± 0.1	0.5 ± 0.3	0.1 ± 0.1	0.2 ± 0.0	0.4 ± 0.0	0.1 ± 0.1	0.2 ± 0.0	0.1 ± 0.0
VitispiraneA[a]	0.00 ± 0.00	0.00 ± 0.00	0.00 ± 0.00	0.00 ± 0.00	0.00 ± 0.00	0.10 ± 0.03	0.13 ± 0.00	0.26 ± 0.01	0.00 ± 0.00	0.00 ± 0.00	0.00 ± 0.00	0.00 ± 0.00	0.00 ± 0.00	0.12 ± 0.02	0.19 ± 0.01	0.35 ± 0.02
RieslingAcetal[a]	0.00 ± 0.00	0.00 ± 0.00	0.00 ± 0.00	0.00 ± 0.00	0.00 ± 0.00	0.32 ± 0.15	0.27 ± 0.01	0.39 ± 0.03	0.00 ± 0.00	0.00 ± 0.00	0.00 ± 0.00	0.00 ± 0.00	0.00 ± 0.00	0.37 ± 0.13	0.35 ± 0.03	0.40 ± 0.01
Ethyl cinnamate	0.0 ± 0.0	1.2 ± 0.7	0.6 ± 0.1	0.0 ± 0.0	0.1 ± 0.0	3.0 ± 2.7	0.7 ± 0.2	1.2 ± 0.4	0.0 ± 0.0	0.1 ± 0.1	1.6 ± 0.0	0.0 ± 0.0	0.0 ± 0.0	2.2 ± 2.0	1.0 ± 0.4	1.0 ± 0.3
Linalool	3.3 ± 0.0	3.6 ± 0.4	2.3 ± 0.1	0.8 ± 0.1	13.9 ± 4.6	20.2 ± 0.4	11.9 ± 0.5	2.9 ± 0.1	3.4 ± 0.1	2.6 ± 0.2	1.3 ± 0.1	0.5 ± 0.1	10.1 ± 0.3	15.3 ± 0.1	8.3 ± 1.3	1.7 ± 0.0
α-terpineol	1.2 ± 0.0	3.0 ± 0.2	3.5 ± 0.1	3.4 ± 0.0	4.7 ± 1.6	22.7 ± 1.3	26.9 ± 0.8	25.5 ± 0.1	1.2 ± 0.1	2.6 ± 0.5	3.0 ± 0.1	1.8 ± 0.1	3.8 ± 0.0	26.6 ± 2.6	27.6 ± 1.0	21.7 ± 0.2
β-citronellol	2.7 ± 0.1	2.0 ± 0.4	1.2 ± 0.1	0.3 ± 0.3	6.7 ± 2.3	3.4 ± 0.0	2.2 ± 0.0	0.9 ± 0.1	3.3 ± 0.3	1.9 ± 0.0	1.2 ± 0.0	0.2 ± 0.2	4.6 ± 0.1	3.1 ± 0.3	2.2 ± 0.3	0.7 ± 0.0
Geraniol	5.9 ± 0.5	2.2 ± 0.7	1.3 ± 0.4	0.6 ± 0.6	13.3 ± 4.4	7.0 ± 0.9	3.7 ± 0.7	1.4 ± 0.4	3.9 ± 0.0	1.2 ± 0.1	0.4 ± 0.4	0.0 ± 0.0	6.0 ± 0.4	5.0 ± 0.1	2.9 ± 0.3	0.5 ± 0.5
Guaiacol	0.2 ± 0.0	0.1 ± 0.0	0.1 ± 0.0	0.2 ± 0.1	3.2 ± 0.4	1.9 ± 0.0	3.0 ± 0.5	4.4 ± 0.2	0.2 ± 0.0	0.2 ± 0.1	0.7 ± 0.1	0.2 ± 0.0	2.2 ± 0.7	2.8 ± 0.1	3.6 ± 0.9	5.6 ± 0.2
4-vinylguaiacol	19.2 ± 11.8	13.0 ± 2.4	10.0 ± 2.1	14.9 ± 5.3	787 ± 188	1453 ± 208	2133 ± 204	2657 ± 13	17.5 ± 0.3	19.9 ± 3.8	48.4 ± 25.2	10.7 ± 2.7	582 ± 5	1305 ± 32	1644 ± 54	2113 ± 120
2-6-dimethoxyphenol	0.2 ± 0.2	0.0 ± 0.0	0.0 ± 0.0	0.0 ± 0.0	9.6 ± 1.1	5.0 ± 0.8	10.6 ± 3.1	15.9 ± 0.1	0.8 ± 0.4	0.4 ± 0.4	2.4 ± 0.0	0.0 ± 0.0	8.3 ± 1.6	6.6 ± 1.0	13.5 ± 5.5	19.0 ± 0.3
E-isoeugenol	0.2 ± 0.0	0.0 ± 0.0	0.0 ± 0.0	0.0 ± 0.0	4.1 ± 1.3	3.1 ± 0.4	3.1 ± 0.2	3.2 ± 0.1	0.1 ± 0.0	0.0 ± 0.0	0.0 ± 0.0	0.0 ± 0.0	1.4 ± 0.0	1.8 ± 0.1	1.6 ± 0.1	2.0 ± 0.2
4-vinylphenol	23.0 ± 3.4	18.6 ± 2.9	11.5 ± 1.0	0.0 ± 0.0	403 ± 149	447 ± 128	770 ± 290	560 ± 17	20.5 ± 4.3	25.5 ± 7.9	206 ± 119	0.0 ± 0.0	343 ± 7	331 ± 5	698 ± 277	491 ± 12
Vanillin	1.0 ± 0.3	0.5 ± 0.3	3.2 ± 1.6	2.0 ± 0.0	13.0 ± 4.1	9.3 ± 0.8	25.8 ± 3.2	11.5 ± 11.5	2.7 ± 1.7	1.2 ± 0.3	1.1 ± 1.1	1.2 ± 0.6	22.4 ± 2.1	8.1 ± 0.3	15.4 ± 2.3	20.1 ± 3.2
Acetovanillone	1.5 ± 0.9	0.0 ± 0.0	2.0 ± 0.8	1.2 ± 1.2	212 ± 78	71.7 ± 40.1	172 ± 3	102 ± 60	2.9 ± 2.0	1.0 ± 0.1	0.9 ± 0.9	0.8 ± 0.8	163 ± 1	32.6 ± 2.5	165 ± 3	92.6 ± 60.1

[a] tentatively identified and given as relative area.

Table S4. 4-way ANOVA assessing the effect of the factors: presence or absence of precursors, yeast strain, aging and their interaction on the volatile composition of Riesling synthetic wine.

	**Precursors**	**Yeast**	**Aging**	**Precursors* Yeast**	**Yeast* Aging**
Ethyl acetate	0.000	< 0.0001	n.s.	0.012	n.s.
Isoamyl acetate	0.012	< 0.0001	< 0.0001	< 0.0001	< 0.0001
Ethyl hexanoate	n.s.[a]	< 0.0001	n.s.	0.000	n.s.
Ethyl octanoate	n.s.	< 0.0001	n.s.	0.003	n.s.
Ethyl decanoate	n.s.	< 0.0001	n.s.	0.047	n.s.
Isobutanol	n.s.	< 0.0001	n.s.	n.s.	n.s.
Isoamyl alcohol	n.s.	< 0.0001	n.s.	n.s.	n.s.
Metionol	0.001	< 0.0001	0.003	n.s.	0.007
β-Phenylethanol	n.s.	< 0.0001	0.017	n.s.	n.s.
Ethyl lactate	n.s.	< 0.0001	0.002	n.s.	n.s.
γ-Butyrolactone	n.s.	< 0.0001	< 0.0001	n.s.	0.010
Butyric acid	n.s.	n.s.	n.s.	n.s.	n.s.
Isobutyric acid	< 0.0001	< 0.0001	n.s.	0.000	n.s.
Hexanoic acid	n.s.	< 0.0001	n.s.	< 0.0001	n.s.
Octanoic acid	n.s.	< 0.0001	n.s.	< 0.0001	n.s.
Decanoic acid	n.s.	< 0.0001	n.s.	n.s.	n.s.
Ethyl isobutyrate	0.041	< 0.0001	< 0.0001	n.s.	< 0.0001
Isobutyl acetate	< 0.0001	< 0.0001	< 0.0001	< 0.0001	< 0.0001
Ethyl 2-methylbutyrate	n.s.	< 0.0001	< 0.0001	n.s.	< 0.0001
Phenylethyl acetate	< 0.0001	< 0.0001	< 0.0001	< 0.0001	< 0.0001
γ-nonalactone	0.002	< 0.0001	0.020	n.s.	n.s.
γ-decalactone	n.s.	< 0.0001	0.000	n.s.	n.s.
TDN	< 0.0001	n.s.	< 0.0001	n.s.	n.s.
β-damascenone	< 0.0001	< 0.0001	0.000	< 0.0001	n.s.
Linalool	< 0.0001	0.002	< 0.0001	0.002	n.s.
α-terpineol	< 0.0001	0.004	< 0.0001	0.004	n.s.
β-citronellol	< 0.0001	0.010	< 0.0001	n.s.	n.s.
Geraniol	< 0.0001	0.011	n.s.	0.006	0.035
4-vinylguaiacol	< 0.0001	n.s.	< 0.0001	n.s.	n.s.
4-vinylphenol	< 0.0001	< 0.0001	0.001	< 0.0001	0.008
vanillin	< 0.0001	0.000	0.001	< 0.0001	0.021
acetovanillone	< 0.0001	< 0.0001	n.s.	< 0.0001	n.s.

[a] n.s—not significant. *—interaction between factors.

Table S5. 3-way ANOVA assessing the effect of the factors: presence or absence of precursors, yeast strain, aging and their interaction on the volatile composition of Garnacha synthetic wine.

Pr > F	Precursors	Yeast	Aging	Precursors* Yeast	Precursors* Aging	Yeast* Aging
Ethyl acetate	< 0.0001	0.00	n.s	n.s	0.02	n.s
Isoamyl acetate	n.s[a]	< 0.0001	n.s	n.s	n.s	0.01
Ethyl hexanoate	0.00	< 0.0001	0.00	0.01	0.03	0.02
Ethyl octanoate	0.00	< 0.0001	0.01	0.01	n.s	n.s
Ethyl decanoate	0.01	0.00	n.s	n.s	n.s	n.s
Isobutanol	n.s	0.01	0.01	n.s	n.s	n.s
1-Butanol	n.s	0.00	0.05	n.s	n.s	n.s
Isoamyl alcohol	0.01	< 0.0001	n.s	n.s	n.s	n.s
1-Hexanol	< 0.0001	< 0.0001	0.00	0.00	0.01	n.s
Metionol	0.01	< 0.0001	< 0.0001	n.s	n.s	n.s
β-Phenylethanol	n.s	< 0.0001	n.s	0.04	n.s	n.s
Ethyl lactate	0.04	< 0.0001	< 0.0001	0.02	n.s	< 0.0001
γ-Butyrolactone	0.00	< 0.0001	< 0.0001	0.01	n.s	< 0.0001
Butyric acid	n.s	< 0.0001	0.00	n.s	n.s	0.00
Isobutyric acid	0.05	< 0.0001	n.s	n.s	n.s	n.s
Hexanoic acid	n.s	< 0.0001	0.01	n.s	n.s	n.s
Octanoic acid	n.s	n.s	n.s	n.s	n.s	n.s
Decanoic acid	0.01	< 0.0001	0.01	0.00	n.s	0.02
Ethyl isobutyrate	0.01	< 0.0001	< 0.0001	n.s	n.s	0.00
Isobutyl acetate	0.05	< 0.0001	0.00	0.04	n.s	0.01
Ethyl 2-methylbutyrate	0.00	< 0.0001	< 0.0001	0.03	0.05	0.01
Ethyl isovalerate	0.00	< 0.0001	< 0.0001	0.01	0.05	< 0.0001
Phenylethyl acetate	0.01	< 0.0001	< 0.0001	0.00	n.s	< 0.0001
γ-nonalactone	0.01	0.01	n.s	n.s	n.s	0.05
γ-decalactone	n.s	< 0.0001	n.s	n.s	n.s	n.s
TDN	< 0.0001	n.s	< 0.0001	n.s	< 0.0001	n.s
β-damascenone	< 0.0001	n.s	0.00	n.s	0.00	n.s
Linalool	< 0.0001	n.s	< 0.0001	n.s	< 0.0001	n.s
α-terpineol	< 0.0001	n.s	< 0.0001	n.s	< 0.0001	n.s
β-citronellol	< 0.0001	n.s	< 0.0001	n.s	0.03	n.s
Geraniol	< 0.0001	0.01	0.00	n.s	n.s	0.03
Guaiacol	< 0.0001	0.01	0.00	0.01	0.00	n.s
4-vinylguaiacol	< 0.0001	0.04	< 0.0001	0.03	< 0.0001	n.s
2-6-dimethoxyphenol	< 0.0001	0.02	0.00	0.03	0.00	n.s
E-isoeugenol	< 0.0001	0.00	n.s	0.00	n.s	n.s
4-vinylphenol	< 0.0001	n.s	0.00	n.s	0.01	n.s
vanillin	< 0.0001	n.s	n.s	n.s	n.s	n.s
acetovanillone	< 0.0001	n.s	n.s	n.s	n.s	n.s

[a] n.s—not significant. *—interaction between factors.

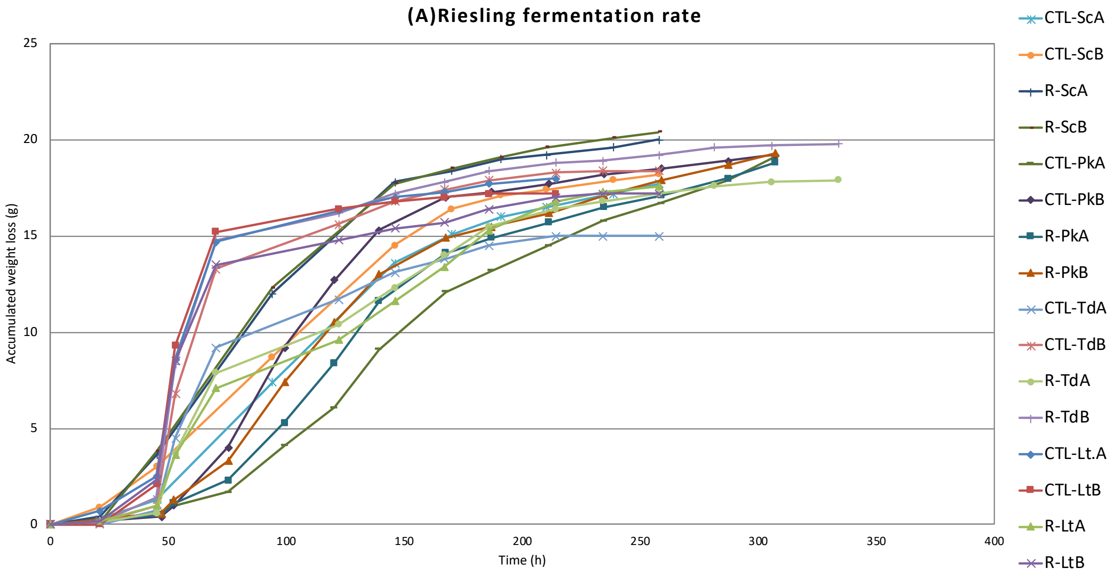

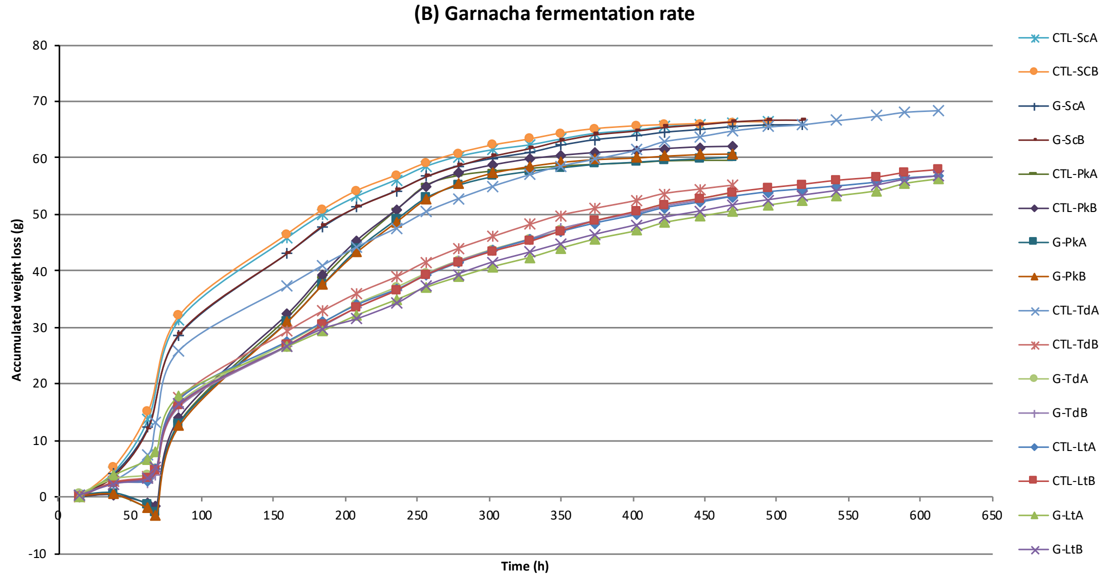

Figure S1. Fermentation rate: Riesling (**A**) and Garnacha (**B**) fermentation rate by means of accumulated weight loss.
